# Evaluation of polyhexamethylene guanidine-induced lung injuries by chest CT, pathologic examination, and RNA sequencing in a rat model

**DOI:** 10.1038/s41598-021-85662-z

**Published:** 2021-03-18

**Authors:** Cherry Kim, Sang Hoon Jeong, Jaeyoung Kim, Ki Yeol Lee, Jaehyung Cha, Chang Hyun Lee, Eun-Kee Park, Ju-Han Lee

**Affiliations:** 1 Department of Radiology, Ansan Hospital, Korea University College of Medicine, 123, Jeokgeum-ro, Danwon-gu, Ansan-si, Gyeonggi 15355 South Korea; 2 Research Institute for Skin Image, Korea University College of Medicine, 123, Jeokgeum-ro, Danwon-gu, Ansan-si, Gyeonggi 15355 South Korea; 3 Medical Science Research Center, Ansan Hospital, Korea University College of Medicine, 123, Jeokgeum-ro, Danwon-gu, Ansan-si, Gyeonggi 15355 South Korea; 4 Department of Radiology, College of Medicine, Seoul National University, Seoul National University Hospital, Seoul, 03080 South Korea; 5 Department of Medical Humanities and Social Medicine, College of Medicine, Kosin University, Busan, 49267 South Korea; 6 Department of Pathology, Ansan Hospital, Korea University College of Medicine, 123, Jeokgeum-ro, Danwon-gu, Ansan-si, Gyeonggi 15355 South Korea

**Keywords:** Diseases, Health care, Medical research

## Abstract

Our aim was to correlate chest CT and pathologic findings of polyhexamethylene guanidine phosphate (PHMG)-induced lung injuries in a rat model, to determine whether PHMG exposure causes lung tumors, and to explore genetic alterations according to PHMG exposure under the guidance of CT. A PHMG solution was intratracheally administrated to 40 male rats. Chest CT was carried out in all rats and both lungs were collected for histopathologic evaluation. At 4- and 8-weeks post-instillation, one lobe of the right lung from 3 rats was subjected to RNA sequencing. At least one abnormal CT finding was found in all rats at all weeks. The major CT findings were inflammation, fibrosis, and tumors in the pathologic analysis, where significant changes were observed over time. The lung lesions remained persistent after 8 weeks of PHMG exposure. In the pathologic analysis, the extent/severity of inflammation did not show statistically significant changes over time, whereas the extent/severity of fibrosis increased continuously up to 6 weeks after PHMG exposure and then decreased significantly at 8 weeks. Bronchiolar-alveolar adenomas which have malignant potential were found in 50% of rats at 6 and 8 weeks after PHMG exposure. Also, several genes associated with lung cancer, acute lung injury, and pulmonary fibrosis were detected. Our study revealed that PHMG-induced lung injury and its changes according to the number of weeks after exposure were demonstrated using chest CT and pathologic evaluation. In addition, we showed that PHMG exposure caused lung tumors and genetic alterations according to PHMG exposure under the guidance of CT.

## Introduction

Polyhexamethylene guanidine phosphate (PHMG) is a member of the polymeric guanidine family which is widely used as a biocide in the medicine, agriculture, and food industries because of its broad-spectrum antibacterial, antifungal, and antiviral activities in addition to its relatively low toxicity for humans^1^. This compound was used as humidifier disinfectants, and several epidemiological and experimental studies revealed a causal association between PHMG exposure through humidifiers and severe lung injuries^2,3^.

In previous studies, chest CT analysis and pathologic correlations of humidifier disinfectant-associated lung disease were performed in both children and adults^4,5^. However, in these studies, only patients who had rapidly progressive respiratory distress for several days or weeks were included. Also, the exact length of time and amount of exposure to the humidifier disinfectant and the exact ingredients of humidifier disinfectants were not known due to the retrospective nature of the study design. In addition, lung biopsies were performed in only a few patients and the percentage of patients exposed to humidifier disinfectant which caused lung injury was not investigated. Also, since PHMG is also known to cause cell cycle arrest and apoptosis in lung epithelial cells^6^, we hypothesized that exposure of PHMG may be related to tumorigenesis.

Genomic responses to PHMG exposure using a DNA microarray was investigated, which has been widely used to simultaneously measure the expression levels of a large numbers of genes^7^. This study showed the PHMG changed the expression of genes involved in the urea cycle, inflammation, and oxidative stress in a lung rat model. However, the authors observed changes of the gene expression without knowing any pathologic changes in rat lungs exposed to PHMG. Thus, it is necessary to confirm the presence of pathologic changes in rat lungs using chest CT before performing DNA microarrays.

Therefore, the purposes of this study were to correlate chest CT and pathologic findings of PHMG-induced lung injury in a rat model, to determine whether PHMG exposure causes lung tumors, and to explore genetic alterations according to PHMG exposure under the guidance of CT.

## Results

### CT image analysis

At least one abnormal CT finding was observed in all rats at all weeks. The CT findings according to weeks after PHMG exposure are shown in Table 1. Consolidation was the most frequent at 2 weeks after PHMG exposure with statistically significance (*p* = 0.012) and then the frequency decreased. Ground-glass opacity (GGO) was observed in all rats (100%) 1, 4, 6, and 8 weeks after PHMG exposure, and in 7 of 8 rats (87.5%) after 2 weeks. Nodules, masses, and linear densities significantly increased according to the number of weeks after PHMG exposure (all P-values for trend < 0.05).

**Table 1:** CT findings and changes according to the groups.

	After 1 week (Group 1)	After 2 weeks (Group 2)	After 4 weeks (Group 3)	After 6 weeks (Group 4)	After 8 weeks (Group 5)	*p* Value	*p* Value for trend
Consolidation	1 (12.5%)	7 (87.5%)	6 (75%)	6 (75%)	3 (37.5%)	0.012	0.767
GGO	8 (100%)	7 (87.5%)	8 (100%)	8 (100%)	8 (100%)	0.395	0.390
Centrilobular nodules	6 (75%)	4 (50%)	7 (87.5%)	4 (50%)	3 (37.5%)	0.229	0.179
Nodule	3 (37.5%)	5 (62.5%)	7 (87.5%)	7 (87.5%)	8 (100%)	0.031	0.003
Mass	0	0	0	1 (12.5%)	2 (25%)	0.217	0.030
Bronchiectasis	0	6 (75%)	8 (100%)	8 (100%)	3 (37.5%)	< 0.001	0.166
Linear densities	0	0	4 (50%)	7 (87.5%)	6 (75%)	< 0.001	< 0.001

GGO, ground glass opacity.

**Figure 1:**
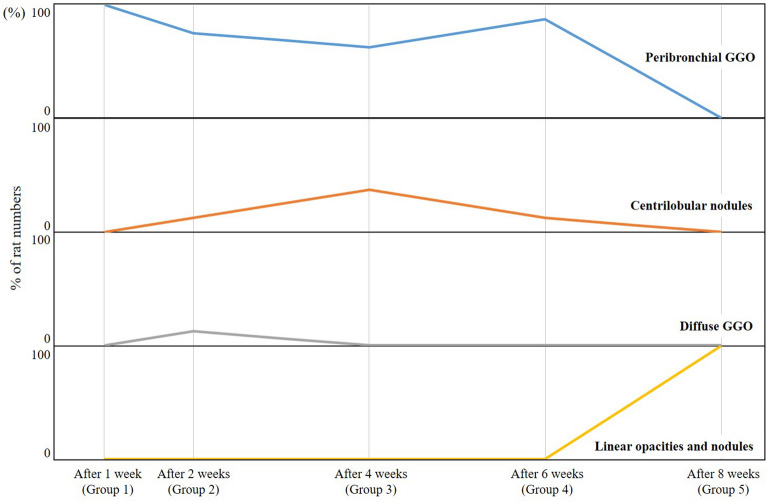
Changes of the CT findings according to the groups. Peribronchial GGO was observed in all rats 1 week after PHMG exposure, then slightly decreased through 2 weeks to 6 weeks after PHMG exposure, and disappeared at 8 weeks after PHMG exposure. Centrilobular nodules peaked at 4 weeks (3 of 8 rats, 37.5%) and then decreased. Linear densities and nodules were observed at 8 weeks (8 of 8 rats, 100%).

The major CT findings, zonal predominance, and prominent location of the CT findings according to the number of weeks after PHMG exposure are shown in Table 2, whereas the changes in the major CT findings according to groups are presented in Fig. 1. Peribronchial GGO was observed in all rats 1 week after PHMG exposure and then slightly decreased from 2 to 6 weeks after PHMG exposure before finally disappearing 8 weeks after PHMG exposure. Centrilobular nodules peaked at 4 weeks (37.5%) and then decreased. Linear densities and nodules were observed at 8 weeks (100%). The major CT findings significantly changed according to the number of weeks (P-value for trend < 0.001). Nearly all lung lesions appeared along the peribronchial area in all weeks.

**Table 2:** Major CT findings, zonal predominance, and prominent location of CT findings according to the groups.

	After 1 week (Group 1)	After 2 weeks (Group 2)	After 4 weeks (Group 3)	After 6 weeks (Group 4)	After 8 weeks (Group 5)	*p* Value	*p* Value for trend
**Major CT findings**							
Peribronchial GGO	8 (100%)	6 (75%)	5 (62.5%)	7 (87.5%)	0	< 0.001	< 0.001
Centrilobular nodules	0	1 (12.5%)	3 (37.5%)	1 (12.5%)	0		
Diffuse GGO	0	1 (12.5%)	0	0	0		
Linear densities and nodules	0	0	0	0	8 (100%)		
**Zonal predominance**							
Upper (above carina)	0	2 (25%)	0	0	0	0.2	0.2
Lower (below carina)	1 (12.5%)	1 (12.5%)	0	0	1 (12.5%)		
Whole lung	7 (87.5%)	5 (62.5%)	8 (100%)	8 (100%)	7 (87.5%)		
**Prominent location**							
Posterior	0	1 (12.5%)	0	0	0	0.4	0.4
Peribronchial	8 (100%)	7 (87.5%)	8 (100%)	8 (100%)	8 (100%)		

GGO, ground glass opacity.

The two radiologists were in good agreement regarding the presence of CT parameters (Cohen’s kappa for GGO, centrilobular nodules, bronchiectasis, and prominent CT features, 1.0; Cohen’s kappa for consolidation, 0.955; Cohen’s kappa for nodules, 0.945; and Cohen’s kappa for mass, 0.877).

### Histologic analysis and western blotting

**Figure 2:**
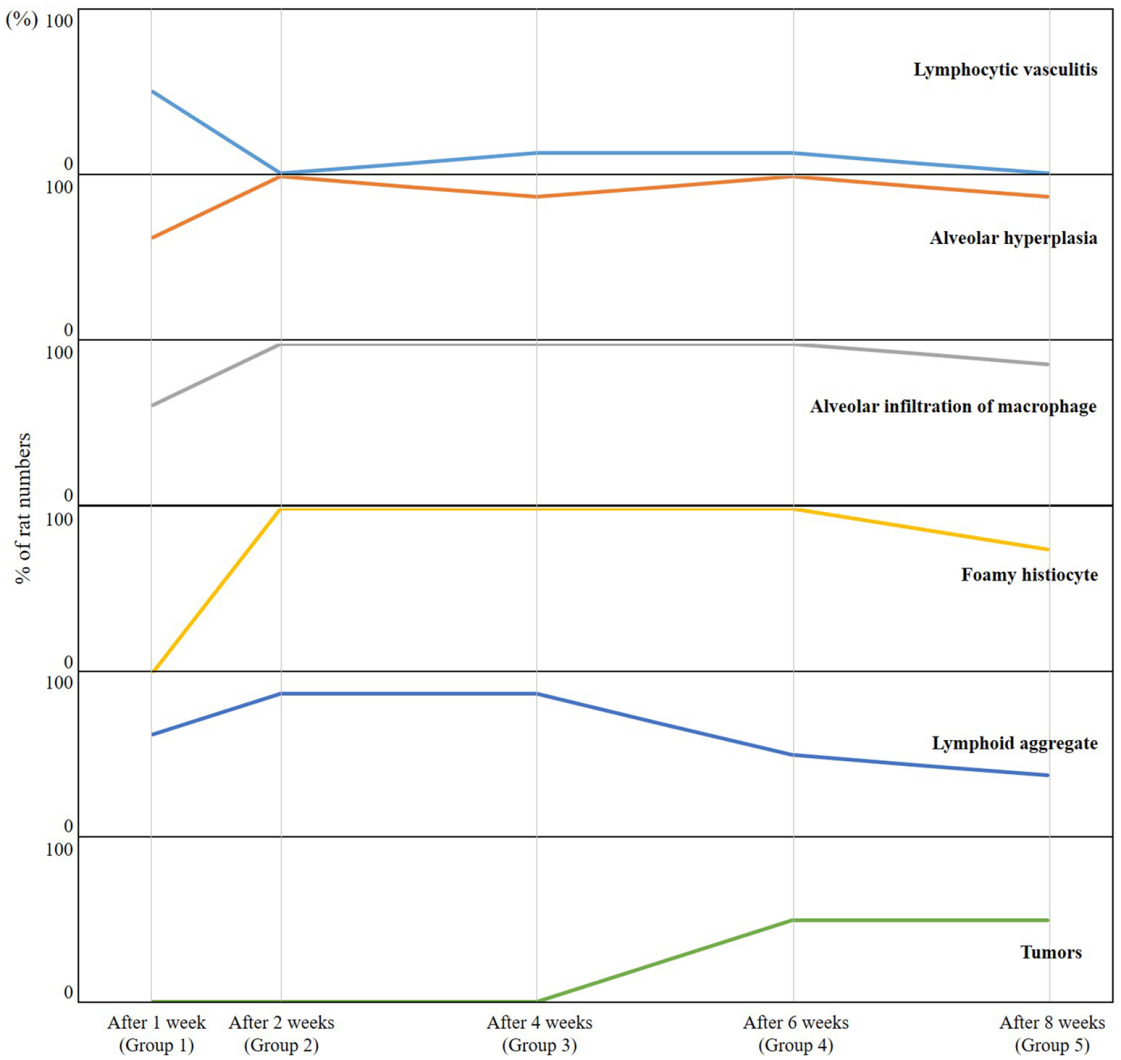
Changes in the pathologic findings according to group. Lymphocytic vasculitis was prominent 1 week after PHMG exposure and then decreased (*p* = 0.057, P-value for trend = 0.028). Alveolar hyperplasia peaked at 6 weeks after PHMG exposure and then decreased (*p* = 0.001, P-value for trend = 0.034). Alveolar infiltration of macrophages was observed continuously over all weeks (*p* = 0.078, P-value for trend = 0.884). Foamy histiocyte and lymphoid aggregate peaked at 4 weeks after PHMG exposure and then decreased (all *p* < 0.05, all P-value for trend > 0.05). Tumors were found in 50% of rats (4 of 8 rats) at 6 weeks and 50% of rats (4 of 8 rats) at 8 weeks after PHMG exposure (all *p* < 0.001, all P-value for trend < 0.001).

The changes in the pathologic findings are shown in Table 3 and Fig. 2. Lymphocytic vasculitis was prominent 1 week after PHMG exposure and then decreased (P-value for trend = 0.028). Alveolar hyperplasia peaked at 6 weeks after PHMG exposure and then decreased (P-value for trend = 0.034). Alveolar infiltration of macrophage was observed continuously over all weeks (P-value for trend = 0.884). The presence of foamy histiocytes and lymphoid aggregates peaked at 4 weeks after PHMG exposure and then decreased (all P-values for trend > 0.05). Tumors were found in 50% of rats at 6 weeks and 50% of rats at 8 weeks after PHMG exposure (all P-values for trend < 0.001). The range of the tumor size was 3 to 8 mm. The tumors were all bronchiolar-alveolar adenomas. The pathologic findings are detailed in Supplementary Figure S1.

**Table 3:** The changes of pathologic findings according to the groups.

	After 1 week (Group 1)	After 2 weeks (Group 2)	After 4 weeks (Group 3)	After 6 weeks (Group 4)	After 8 weeks (Group 5)	*p* Value	*p* Value for trend
**Lymphocytic vasculitis**							
None	4 (50%)	8 (100%)	7 (87.5%)	7 (87.5%)	8 (100%)	0.057	0.028
Mild	1 (12.5%)	0	0	1 (12.5%)	0		
Moderate	0	0	1 (12.5%)	0	0		
Severe	3 (37.5%)	0	0	0	0		
**Alveolar hyperplasia**							
None	3 (37.5%)	0	0	0	1 (12.5%)	0.001	0.034
Mild	3 (37.5%)	3 (37.5%)	1 (12.5%)	0	2 (25%)		
Moderate	2 (25%)	4 (50%)	1 (12.5%)	0	4 (50%)		
Severe	0	1 (12.5%)	6 (75%)	8 (100%)	1 (12.5%)		
**Alveolar infiltration of macrophage**							
None	0	0	0	0	0	0.078	0.884
Mild	3 (37.5%)	2 (25%)	0	0	1 (12.5%)		
Moderate	3 (37.5%)	1 (12.5%)	5 (62.5%)	7 (87.5%)	5 (62.5%)		
Severe	2 (25%)	5 (62.5%)	3 (37.5%)	1 (12.5%)	2 (25%)		
**Foamy histiocyte**							
None	8 (100%)	0	0	0	2 (25%)	< 0.001	0.058
Mild	0	2 (25%)	0	2 (25%)	1 (12.5%)		
Moderate	0	5 (62.5%)	4 (50%)	5 (62.5%)	5 (62.5%)		
Severe	0	1 (12.5%)	4 (50%)	1 (12.5%)	0		
**Lymphoid aggregate**							
None	3 (37.5%)	1 (12.5%)	1 (12.5%)	4 (50%)	5 (62.5%)	0.016	0.239
Mild	4 (50%)	7 (87.5%)	2 (25%)	1 (12.5%)	3 (37.5%)		
Moderate	0	0	4 (50%)	3 (37.5%)	0		
Severe	1 (12.5%)	0	1 (12.5%)	0	0		
**Tumors**							
Presence of tumors	0	0	0	4 (50%)	4 (50%)	< 0.001	< 0.001
Number of tumors	0	0	0	1.6 ± 3.1	3.4 ± 8.3	< 0.001	< 0.001

The extent and severity of inflammation and fibrosis are shown in Table 4 and Supplementary Figure S2. The extent and severity of inflammation were observed continuously throughout all weeks without statistically significant changes (all P-values for trend > 0.05). There were no significant changes between the weeks in the inflammation scores (P-value for the trend = 0.82). The extent and severity of inflammation and fibrosis gradually increased up to 6 weeks after PHMG exposure and then decreased at 8 weeks (all P-values for trend < 0.05). The fibrosis scores were also significantly greatest at 6 weeks after PHMG exposure and then decreased at 8 weeks (P-value for trend = 0.007). The expression of myofibroblast proteins markers, such as fibronectin, collagen type I, and α-SMA, was detected by western blotting at 4, 6, and 8 weeks post-PHMG exposure (Supplementary Figure S3).

**Table 4:** The extent and severity of inflammation and fibrosis according to the groups.

	After 1 week (Group 1)	After 2 weeks (Group 2)	After 4 weeks (Group 3)	After 6 weeks (Group 4)	After 8 weeks (Group 5)	P-value	P-value for trend
**Inflammation extent**							
None	0	0	0	0	0	0.075	0.932
< 0–25%	4 (50%)	0	0	0	2 (25%)		
< 25–50%	3 (37.5%)	6 (75%)	6 (75%)	6 (75%)	6 (75%)		
> 50%	1 (12.5%)	2 (25%)	2 (25%)	2 (25%)	0		
**Inflammation severity**							
None	0	0	0	0	0	0.312	0.209
Mild	0	0	0	0	1 (12.5%)		
Moderate	6 (75%)	3 (37.5%)	3 (37.5%)	6 (75%)	5 (62.5%)		
Severe	2 (25%)	5 (62.5%)	5 (62.5%)	2 (25%)	2 (25%)		
Inflammation score	3.88 ± 1.13	4.88 ± 0.84	4.88 ± 0.84	4.50 ± 0.93	3.88 ± 0.99	0.087	0.82
**Fibrosis extent**							
None	5 (62.5%)	2 (25%)	0	0	1 (12.5%)	0.007	0.024
< 0–25%	3 (37.5%)	6 (75%)	6 (75%)	3 (37.5%)	7 (87.5%)		
< 25–50%	0	0	1 (12.5%)	2 (25%)	0		
> 50%	0	0	1 (12.5%)	3 (37.5%)	0		
**Fibrosis severity**							
None	5 (62.5%)	2 (25%)	0	0	1 (12.5%)	0.014	0.014
Mild	2 (25%)	4 (50%)	5 (62.5%)	1 (12.5%)	4 (50%)		
Moderate	1 (12.5%)	2 (25%)	1 (12.5%)	4 (50%)	3 (37.5%)		
Severe	0	0	2 (25%)	3 (37.5%)	0		
Fibrosis score	0.88 ± 1.25	1.75 ± 1.17	3.00 ± 1.60	4.25 ± 1.58	2.13 ± 0.99	0.002	0.007

### Radiologic-histologic correlation

**Figure 3:**
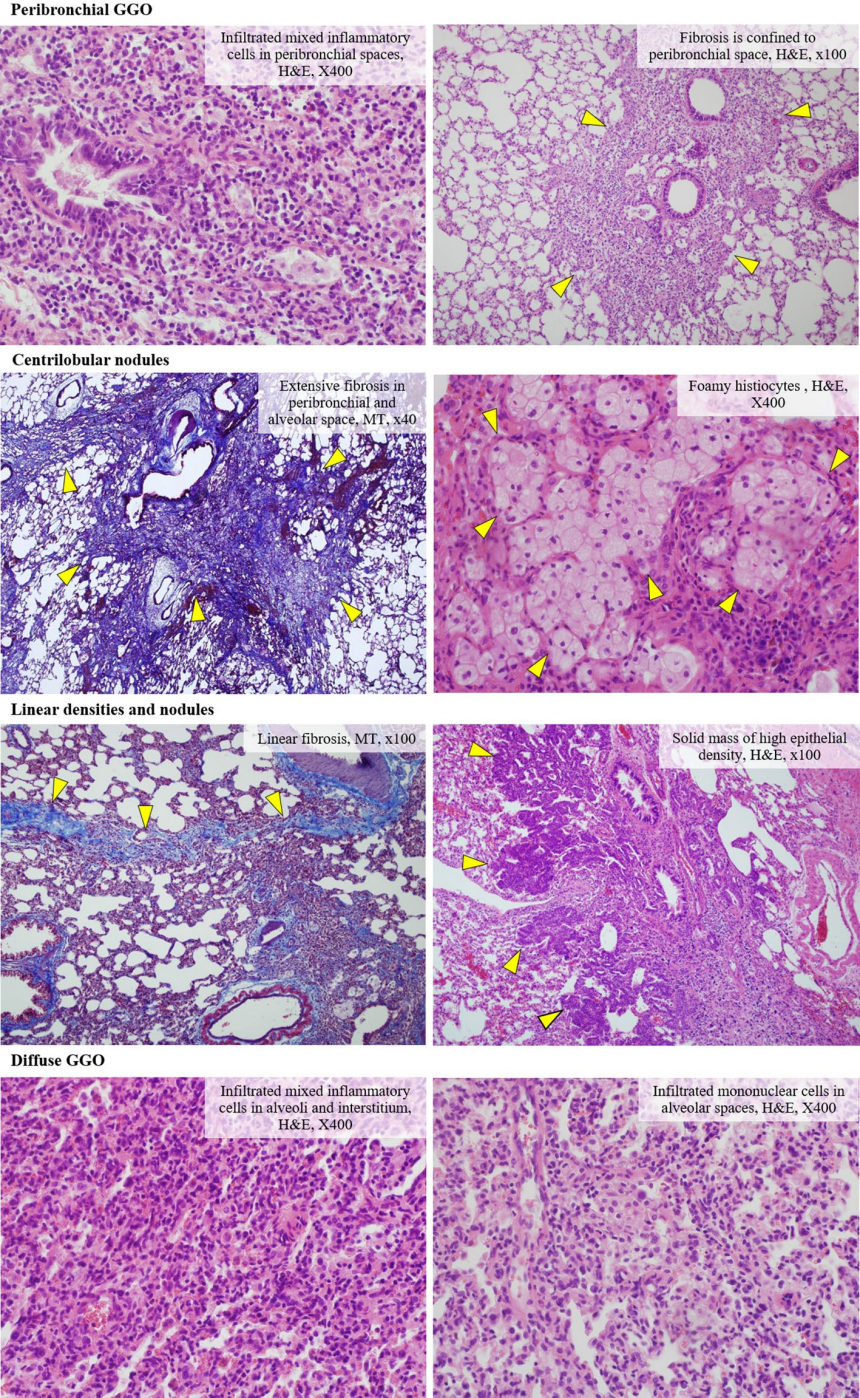
Four major CT findings and matched major histologic findings. Through radiologic-histologic correlation, 84.6% of peribronchial GGOs were inflammation and the rest were fibrosis. Centrilobular nodules were 60% fibrosis and the rest were inflammation. Linear densities and nodules were 62.5% inflammation, 25% fibrosis, and 12.5% tumors, and diffuse GGO was inflammation (100%).

Four major CT findings and the matched major and minor histologic findings are shown in Fig. 3 and Supplementary Table S1. Peribronchial GGO was observed in 26 rats (65%) in all groups, and was matched with the infiltrate of histiocytes (84.6%) and fibrosis in peribronchial and/or alveolar spaces (15.4%). Centrilobular nodules were found in 5 rats in all groups (12.5%) in addition to matched fibrosis in peribronchial and/or alveolar spaces (60%) and the infiltrate of histiocytes and lymphocytes in the peribronchial/alveolar space (40%). Linear densities and nodules were major findings of 8 rats (20%) in Group 5. The matched major pathologic findings were the infiltrate of histiocytes and lymphocytes in peribronchial/alveolar spaces (62.5%), fibrosis in peribronchial/alveolar spaces (25%), and bronchiolo-alveolar adenomas (12.5%). Diffuse GGO was found in one rat in Group 2 and the matched histologic finding was the infiltrate of histiocytes in the alveolar spaces.

### RNA sequencing analysis

**Figure 4:**
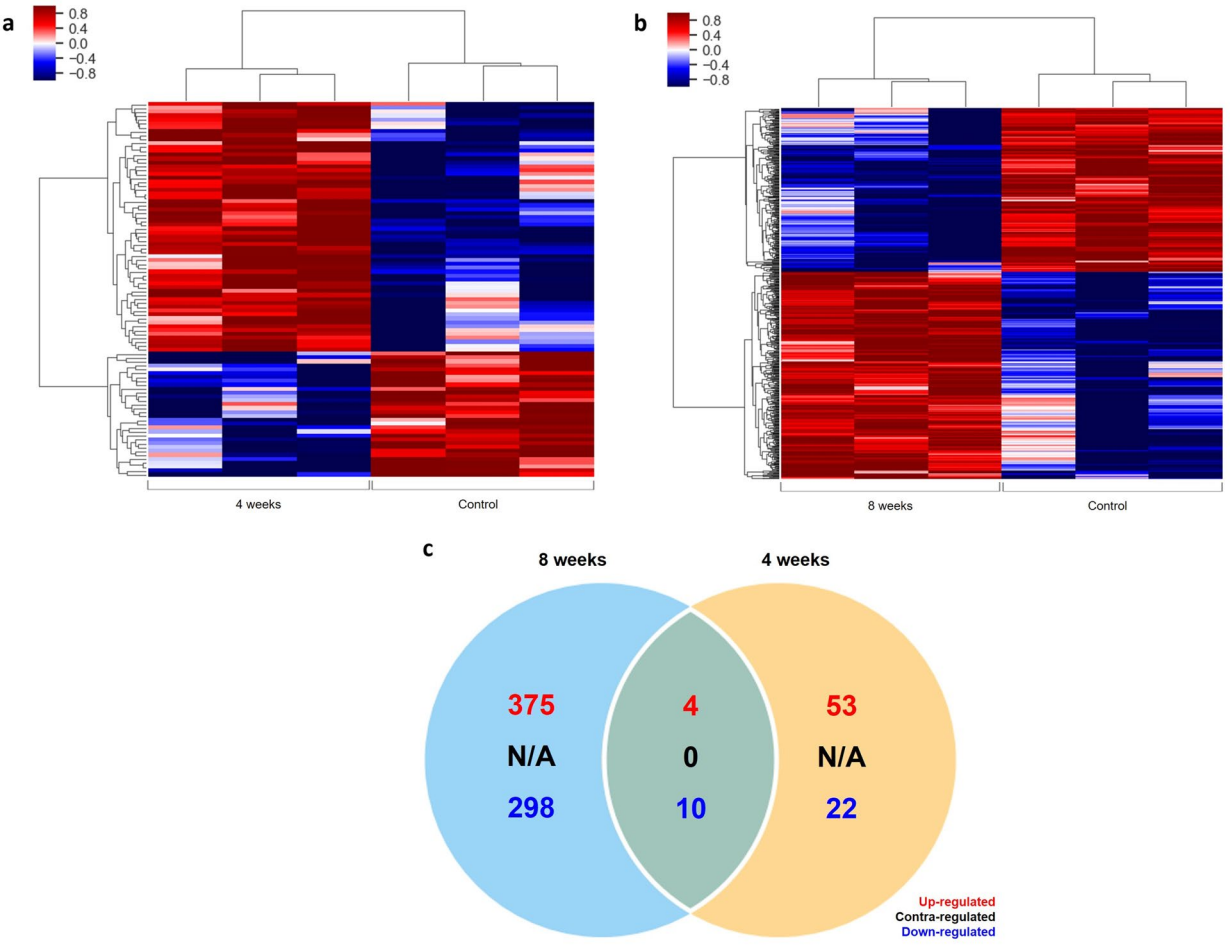
Analysis of PHMG-regulated gene expression in rat lung tissue. Heatmaps of PHMG regulated genes at 4 and 8 weeks post-PHMG exposure (frames **a** and **b**). A Venn diagram shows the numbers of genes that were up-regulated, contra-regulated, and down-regulated between 4 and 8 weeks after PHMG exposure (frame **c**).

In the clustering heatmap, the total number of PHMG-related genes were increased at 8 weeks compared with 4 weeks after PHMG exposure (Fig. 4). In addition, 375 up-regulated and 298 down-regulated genes among 17,048 genes in lung tissues were newly detected 8 weeks post-PHMG exposure. Supplementary Tables S2 and S3 summarize the genes significantly up-regulated and down-regulated due to PHMG exposure. At 4 weeks after PHMG exposure, there were some upregulated genes that have been implicated in mediating pulmonary disorders, such as *ALOX15*, which induces acute lung injury^8^, and *PDE1A*, *CHI3L1*, and *BPIFB1*, which play a critical role in pulmonary fibrosis^9–11^. However, those genes were not detected at 8 weeks after PHMG exposure. Meanwhile, at 8 weeks after PHMG exposure, lung cancer-related genes such as *TOP2A* and *MKI67* and tumor metastasis-related genes such as *CDH11* and *CD44* were significantly upregulated^12–15^.

Gene Ontology enrichment analysis showed genes related to apoptotic processes, cell differentiation, the extracellular matrix, and immune and inflammatory responses (Supplementary Figure S4). Interestingly, a number of genes related to DNA repair and RNA splicing were not expressed at 4 weeks but were increased 8 weeks after PHMG exposure.

## Discussion

In this study, we evaluated PHMG-induced lung injury and its changes according to the number of weeks after exposure in a rat model using chest CT and pathologic evaluation. In addition, we proved that PHMG exposure caused lung tumors and genetic alterations under the guidance of CT.

In both the chest CT and pathologic analyses, at least one lesion in the lung appeared every week in all rats exposed to PHMG, despite the single exposure. In addition, the major CT findings of lung lesions showed significant changes over time, which were also proved though pathologic evaluation, and the lung lesions remained persistent after 8 weeks of exposure. In the pathologic analysis, the extent and severity of inflammation did not show statistically significant changes over time, whereas the extent and severity of fibrosis increased continuously up to 6 weeks after exposure and then decreased significantly at 8 weeks. Among the major CT findings, we found that 84.6% of peribronchial GGOs were inflammation and the rest were fibrosis through a radiologic-histologic correlation. Centrilobular nodules were 60% fibrosis and the rest were inflammation. The linear densities and nodules were 62.5% inflammation, 25% fibrosis, and 12.5% tumors, and diffuse GGO was inflammation (100%). Most of the lesions were located along the peribronchial area, which may be due to the fact that PHMG was instilled through the trachea and reacted by spreading along the bronchus.

These findings suggest that PHMG can cause significant lung injury and if exposed to PHMG, the lesion can be evaluated by chest CT. In previous studies, exudates fill the alveolar air space as well as the peribronchial fibro-inflammatory lesions in both early and chronic stages in pediatric patients^16^. In adult patients, extensive fibrosis was also noted in the chronic stage^5^. However, in the previous study, they did not analyze pathologic findings which correlated with CT findings in all patients. Several studies using mice also reported severe pulmonary inflammation and fibrosis caused by PHMG exposure^17,18^. PHMG exposure led to persistent pulmonary inflammation and fibrosis for at least 10 weeks and dose-dependent exacerbation of both inflammation and pulmonary fibrosis on day 14 was found. However, these studies did not provide quantitative pathologic finding results and did not analyze the changes of pathologic findings over time in detail. In addition, there have been no studies on the occurrence of lung lesions and changes according to time caused by PHMG using chest CT.

There have been several studies regarding drugs or chemicals, such as bleomycin, hydrochloric acid (HCL), and nitrogen mustard (NM), inducing pulmonary fibrosis in the rodent model. Bleomycin is the drug best characterized for pulmonary fibrosis in the rodent model. The development of pulmonary fibrosis by bleomycin occurs around 3–4 weeks after a single dose with a relatively quick peak fibrotic response^19^. However, the bleomycin model possesses the self-limiting nature of fibrosis, and the fibrotic lesions reportedly resolved after 3–4 weeks^20,21^. Tracheal administration of HCL is a well-established model to study acute lung injury^22,23^, and a recent report revealed that HCL also induced pulmonary fibrosis about 4 weeks (30 days) after HCL instillation^24^. However, there are currently no reports regarding chronic pulmonary fibrosis for more than 4 weeks. NM is also a cytotoxic vesicant that induces lung injury and fibrosis^25,26^. However, these changes in a rodent’s lung due were observed for up to 4 weeks, with no known change after that time. In our study, PHMG caused very strong and persistent lung injury in 8 weeks, which is a significantly different lung injury than those caused by other drugs/chemicals.

Another important finding in our study is the incidence of tumors caused by PHMG. Previous studies have not reported the incidence of tumors, probably because the pathologic evaluation did not include the section where the tumor grew. In this study, the CT findings were analyzed in advance and slides were made in consideration of the mass or nodule part. As a result, we found tumors in 50% of rats 6 and 8 weeks after exposure. Our study was the first to detect tumors in lungs exposed to PHMG.

In our study, the tumors were all bronchiolar-alveolar adenomas. A spectrum of bronchiolar-alveolar proliferative lesions such as hyperplasia-adenoma-adenocarcinoma has been best described in rodents, where they can occur after exposure of various carcinogens or spontaneously^27^. Bronchiolar-alveolar proliferative lesions apparently represent a spectrum that progresses from hyperplasia to adenoma to carcinoma in rodents and some researchers have argued that all lesions should be designated as carcinomas, even in earliest lesions. In addition, bronchioloalveolar neoplasms in human are generally considered malignant^27^. In our study, analysis was only performed up to 8 weeks after PHMG exposure, but considering the spectrum of bronchiolar-alveolar proliferation, the possibility of carcinoma being discovered after 8 weeks cannot be excluded. Therefore, in future work, it is important to consider results beyond 8 weeks.

In the RNA sequencing analysis, we found several genes associated with lung cancer, acute lung injury, and pulmonary fibrosis. PHMG-related genes were changed and increased at 8 weeks compared with 4 weeks after PHMG exposure. For example, soon after PHMG exposure (i.e., 4 weeks post-PHMG exposure), genes related to acute lung injury and pulmonary fibrosis were detected, and genes associated with lung cancer and tumorigenesis were detected 8 weeks post-PHMG exposure. Furthermore, there were no genes associated with DNA repair and RNA splicing at 4 weeks after PHMG exposure, but such genes were newly detected at 8 weeks, which suggests PHMG may induce cell damage in lung tissue over time. It has previously been reported that dysregulation of DNA repair and RNA splicing can cause various genetic disorders and eventually lead to cancer^28–30^.

Functional classification analysis revealed that lung cancer-related genes, such as *TOP2A* and *MKI67,* and tumor metastasis-related genes, such as *CDH11* and *CD44,* were significantly up-regulated after 8 weeks of PHMG exposure. Among lung cancer genes, *TOP2A* is a nuclear enzyme critical for the regulation of DNA transcription, replication, and recombination^31^. Moreover, it has been shown that TOP2A expression is upregulated in lung adenocarcinoma patients and is associated with a poor prognosis^13^. MKI67 is widely used as a tumor proliferation marker and an important therapeutic target of malignant tumors^14^. In addition, high ki67 expression is found in lung cancer patients^12^. Among tumor metastasis-related genes, CDH is a cell adhesion molecule with a crucial role in the formation of cell–cell adhesion junctions, especially in tumor metastasis. CDH11 and CD44 are over-expressed in oral squamous cell carcinoma (OSCC) and involved in OSCC metastasis^15^.

Taken together, our results demonstrate that genetic alterations due to PHMG exposure may provoke pulmonary inflammation and pulmonary fibrosis by attenuating the normal recovery mechanism of the lung, consequently resulting in tumorigenesis. Furthermore, since the lung lobes with lesions were identified beforehand using CT, we selected the tissues for RNA sequencing, resulting in a more robust and direct gene that was associated with the lung lesions.

There were several limitations in this study. First, RNA sequencing was performed for only three rats, not in all rats in each group. However, because the primary goal of this study was CT imaging analysis with pathologic correlation, much of the tissue could not be utilized for RNA sequencing. Second, it is difficult to accurately correlate the dose instilled in rats with the amount of inhalation through the humidifier in humans, and we did not perform experiments to assess the dose-dependent link between the tumors and PHMG exposure. Further studies are needed to determine the extent and severity of lung lesions, including tumors, using a smaller or higher dose in addition to the concentrations used in this experiment.

In conclusion, at least one lesion in the lung appeared every week in all rats exposed to PHMG in chest CT and pathologic analyses, despite the single exposure. In addition, the major CT findings of lung lesions showed significant changes over time, which were also verified though pathologic evaluation, and the lung lesions remained persistent after 8 weeks of exposure. We found bronchiolar-alveolar adenomas, which have malignant potential, in 50% of rats 6 and 8 weeks after exposure. Also, several genes associated with lung cancer, acute lung injury, and pulmonary fibrosis were found. The genetic alterations due to PHMG exposure may provoke pulmonary inflammation and pulmonary fibrosis by attenuating the normal recovery mechanism of the lung and consequently result in tumorigenesis.

## Methods

This study was approved by the Institutional Animal Care and Use Committee of the Korea University Medical Center (Approval number: 2019–0031). This study was carried out in compliance with the ARRIVE guidelines, and all experiments were also performed in accordance with Korea university guidelines.

### Animals

Nine-week-old male Sprague–Dawley rats (Raonbio, Yong-in, South Korea) were acclimated for 1 week (3 rats per cage) under the following conditions: temperature, 22–25 ℃; relative humidity, 40–60%; and lighting condition, light 12 h/dark 12 h. Pelletized food for experimental rodents (Purina, Sung-nam, South Korea) and filtered tap water were given ad libitum.

### Experimental design

**Figure 5:**
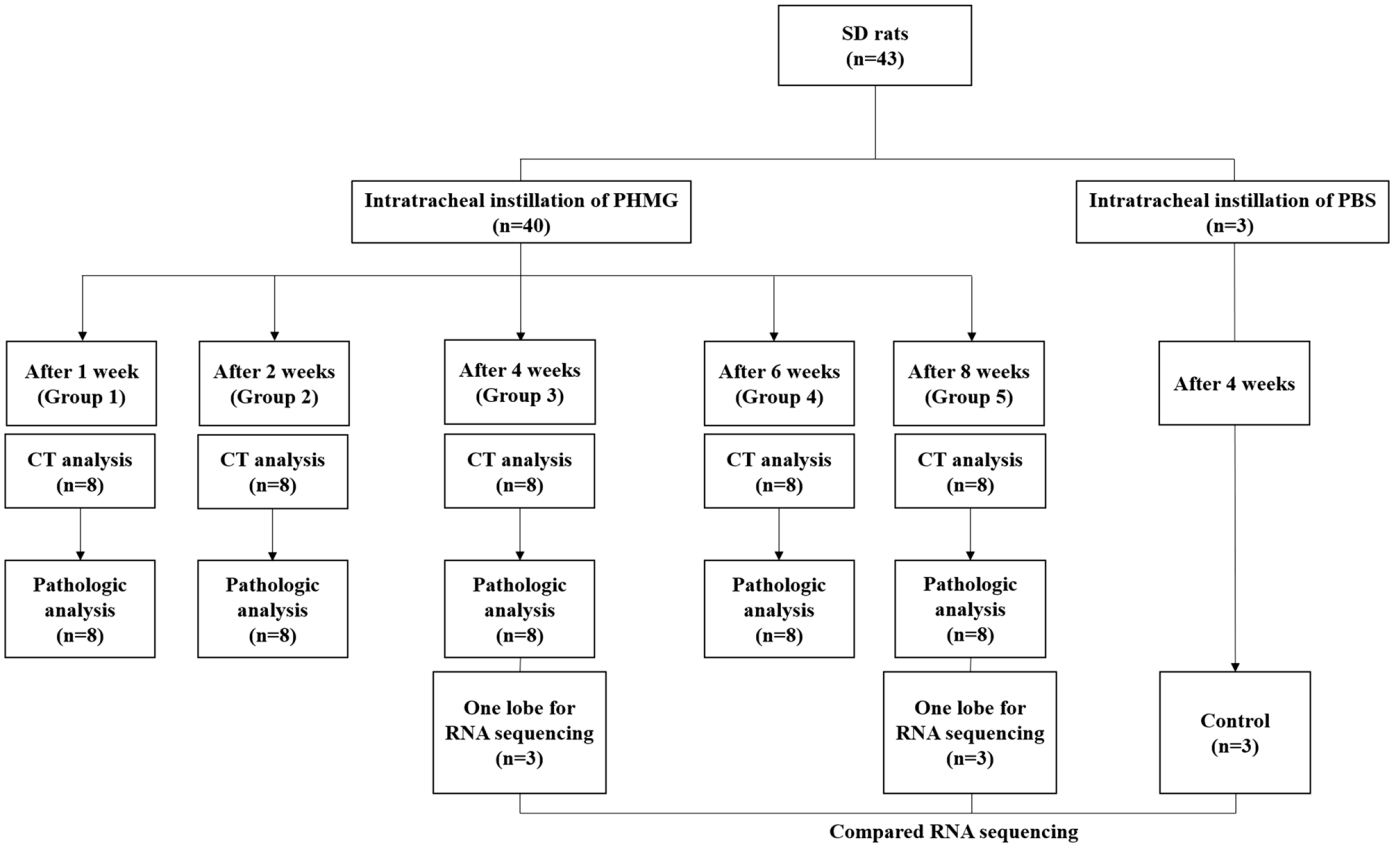
The experimental design. 1, 2, 4, 6, and 8 weeks after instillation (Groups 1 to 5), chest CT examination was conducted in all rats under anesthesia. Subsequently, the animals were sacrificed and both lungs were collected for histopathologic evaluation. In Group 3 and 5 (4 and 8 weeks after instillation), one lobe of the right lung from 3 randomly chosen rats were used for RNA sequencing and the other lobes of those 3 rats were used for histopathologic evaluation. The lung tissue from control animals (n = 3) was also extracted 4 weeks after the instillation of sterile saline instead of PHMG for RNA sequencing.

A total of 40 rats were randomly divided into 5 groups. A solution of PHMG was diluted to 0.9 mg/kg with saline using a previously reported method^18^. Rats were anesthetized with 2% isofluorane in 70% N_2_O and 30% O_2_ for intratracheal instillation of PHMG. Then, 50 uL of the PHMG solution was intratracheally administrated to the rats under the guide of a modified videoscope for intratracheal instillation (Supplementary Figure S5). At weeks 1, 2, 4, 6, and 8 post-instillation (Groups 1 to 5), chest CT examinations were carried out on all rats under anesthetic conditions with an intraperitoneal and intramuscular injection of Alfaxan (30 mg/kg) and Xylazine (10 mg/kg), respectively. Subsequently, the animals were sacrificed and both lungs were collected for histopathologic evaluation. In Groups 3 and 5, one lobe of the right lung from 3 randomly chosen rats were used for RNA sequencing and the rest of other lobes of these 3 rats were used for histopathologic evaluation. The one lobe in each rat used for RNA sequencing was chosen by one radiologist (C.K.) after reviewing the CT images (lobe with obvious lesions). Lung tissues from the control animals (n = 3) were also extracted at 4 weeks after sterile saline instillation for RNA sequencing. The experimental design is summarized in Fig. 5.

### CT protocol

All CT images were scanned using a Philips IQon 128-slice dual-layer detector spectral CT scanner (Philips Healthcare, Cleveland, OH, USA). All images were obtained in a caudo-cranial direction during an inspiration breath-hold using a ventilator for small animals (VentElite, Harvard Apparatus, MA, USA). CT scan parameters were as follows: kVp, 80; mA, 400; collimation, 64 × 0.625 mm; slice thickness, 0.67 mm; beam width, 40 mm; pitch, 1.048; rotation time, 0.4 s.

### CT evaluation

**Figure 6:**
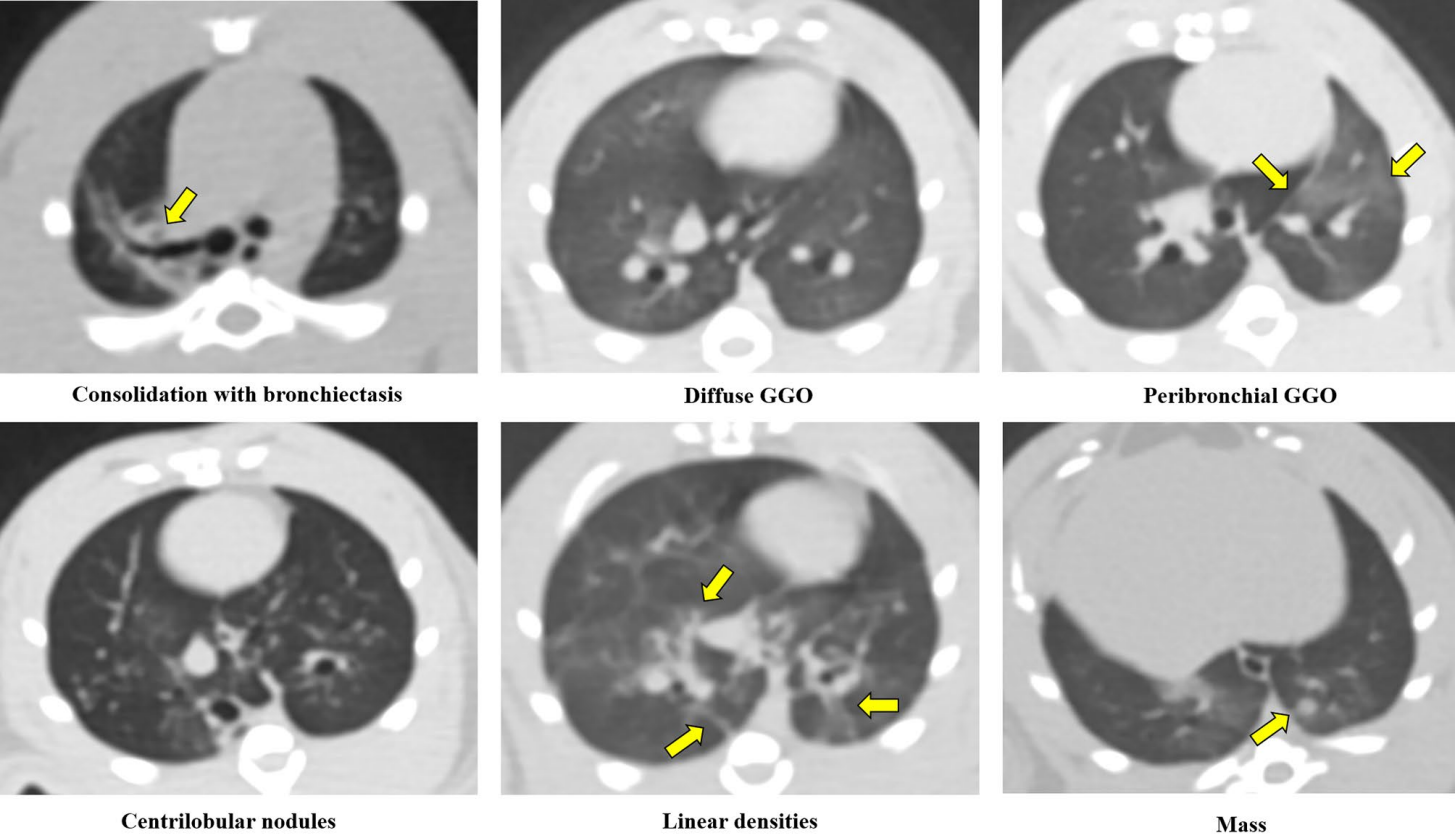
Examples of CT findings. Consolidation, ground-glass opacity (GGO), nodules, masses, centrilobular nodules, bronchiectasis, and linear atelectasis were followed or modified the glossary of radiologic terms suggested by the Fleishner Society.

Two board certified radiologists (K.Y.L. and C.K., with 22 and 10 years of experience in thoracic imaging, respectively) who were blinded to the experimental groups and time points reviewed all CT images. Each reviewer evaluated the following CT findings (Fig. 6). The CT findings followed or modified the glossary of radiologic terms suggested by the Fleishner Society^32^. Consolidation was defined as a homogeneous increase in parenchymal attenuation obscuring margins of vessel and airway walls. Hazy increased lung opacity with the preservation of bronchial and vascular margins was defined as GGO. A nodule was defined as a rounded or irregular opacity, well or poorly defined, measuring up to 1 mm in diameter. A well or poorly defined, rounded or irregular opacity over 1 mm was defined as a mass. Centrilobular nodules were nodules which appeared to be separated from the pleural surfaces, fissures, and interlobular septa. Bronchiectasis included bronchial dilatation with respect to the accompanying pulmonary artery, with a lack of tapering of the bronchi. The linear density was a focal or multifocal subsegmental atelectasis showing linear configuration, almost always extending to the pleura.

Among these CT findings, the findings that make up more than two-thirds of the lesions were defined as major CT findings. There were four major CT findings: peribronchial GGO, centrilobular nodules, diffuse GGO, and linear densities and nodules.

The zonal distribution was considered as being the upper lung zone (above the level of the carina), lower lung zone (below the level carina), or whole lung. The prominent location was defined to be ‘posterior’ if there was a predominance of CT findings in the dorsal area and 'peribronchial' if there was predominance along the peribronchovascular area.

### Histologic examination

All extracted lung specimens were evaluated by one experienced pathologist with 20 years of clinical experience in lung pathology (J.L.). The lungs were fixed in 10% neutral buffered formalin. From the fixed samples, 4 um thick paraffin sections were cut and hematoxylin and then, eosin (H&E) staining and Masson’s trichrome (MT) staining were performed.

The extent (none, lesions involving < 0–25%/ < 25–50%/ > 50% of the total lung areas) and severity (none/mild/moderate/severe) of inflammation and fibrosis were evaluated. The scores of inflammation and fibrosis were calculated by adding the extent and severity of inflammation and fibrosis.

### Radiologic-histologic correlation

All major CT findings were compared with matched histologic findings, lesion by lesion, by one radiologist (C.K.) and one pathologist (J.L.), with consensus.

### RNA isolation

One lobe in each rat (each 3 rats in Group 3, Group 5, and the control group) were selected after the radiologist reviewed the correlated chest CT. The radiologist chose lobes with lesions similar to those of the other lobes. Lobes with no or too few lesions were excluded from consideration. Then, the selected lobes were ground and lysed using lysis buffer containing 2-mercaptoethanol. The total RNA was isolated using Trizol reagent (Invitrogen, Carlsbad, CA). The RNA quality was assessed by an Agilent 2100 Bioanalyzer (Agilent Technologies, Santa Clara, CA, USA) using the RNA 6000 Nano Chip (Agilent Technologies, Amstelveen, The Netherlands) and RNA quantification was performed using a NanoDrop 2000 spectrophotometer (Thermo Scientific, Wilmington, DE, USA).

### Western blotting

Lung tissues at 4, 6, and 8 weeks after PHMG exposure were lysed in T-PER™ Tissue Protein Extraction Reagent (Thermo Scientific, Rockford, IL, USA) using a homogenizer (OMNI International, Waterbury, CT, USA). Equal amounts of protein extracts (20 μg) were separated by sodium dodecyl sulfate–polyacrylamide gel electrophoresis and then transferred onto a polyvinylidene fluoride membrane (Atto, Tokyo, Japan). After blocking with 5% non-fat skim milk for 1 h, the membrane was incubated overnight with rabbit anti-fibronectin (1:1,000, Abcam, Cambridge, UK), rabbit anti-collagen type I (1:1,000, Abcam), mouse anti-α-SMA (1:1,000, Abcam), and mouse β-actin (1:5,000, Santa Cruz, CA, USA) primary antibodies. Afterward, an appropriate horseradish peroxidase-conjugated anti-rabbit IgG or anti-mouse IgG antibody (Cell Signaling Technology, Danvers, MA, USA) was used to bind to the primary antibodies. Protein bands were imaged using the ChemiDoc Touch Imaging System (Bio-Rad Laboratories).

### Library preparation and sequencing

For the control and test RNAs, the construction of the library was performed using a QuantSeq 3′ mRNA-Seq Library Prep Kit (Lexogen, Inc., Austria) according to the manufacturer’s instructions. In brief, 500 ng of the total RNA was prepared and an oligo-dT primer containing an Illumina-compatible sequence at its 5′ end was hybridized to the RNA and reverse transcription was performed. After degradation of the RNA template, second strand synthesis was initiated by a random primer containing an Illumina-compatible linker sequence at its 5′ end. The double-stranded library was purified by using magnetic beads to remove all reaction components. The library was amplified to add the complete adapter sequences required for cluster generation. The finished library was purified from PCR components. High-throughput sequencing was performed as single-end 75 sequencing using a NextSeq 500 (Illumina, Inc., USA).

### Data analysis

QuantSeq 3′ mRNA-Seq reads were aligned using Bowtie2^33^. Bowtie2 indices were either generated from the genome assembly sequence or the representative transcript sequences for aligning to the genome and transcriptome. The alignment file was used for assembling transcripts, estimating their abundances, and detecting differential expression of genes. Differentially expressed genes were determined based on counts from unique and multiple alignments using coverage in Bedtools^34^. The read count (RC) data was processed based on the quantile normalization method using EdgeR within R using Bioconductor^35,36^. The gene lists were further analyzed in the Gene Ontology database to identify expressed genes with similar functions through the online website http://david.niaid.nih.gov using DAVID and Medline databases (http://www.ncbi.nlm.nih.gov/). The genes whose expression significantly changed (standard P-value < 0.05 and log > 2 or < -2) by PHMG exposure were identified.

### Statistical analysis

The chi-square test for nominal variables and Kruskal–Wallis test for continuous variables were performed to determine differences CT features and pathologic findings among groups, and the chi-square trend analysis for nominal data, Cochran–Mantel–Haenszel test for ordinal data, Jonckheere-Terpstra test for continuous data with Bonferroni corrections were performed for revealing the chronologic changes of CT features and pathologic findings. Inter-observer agreement between two radiologists was assessed with Cohen’s kappa statistics. These results were interpreted as follows: < 0.2, poor agreement; 0.21–0.4, fair agreement; 0.41–0.6, moderate agreement; 0.61–0.8, good agreement; > 0.80, very good agreement. All statistical analyses were performed using SPSS Statistics 20 (SPSS, Chicago, IL, USA) or MedCalc version 18.5 (MedCalc Software, Ostend, Belgium). All P-values < 0.05 were considered statistically significant.

## Supplementary Information

Supplementary Files

## Data Availability

All data generated or analysed during this study are included in this published article.
