# Supplementary material for: Evaluation of polyhexamethylene guanidine-induced lung injuries by chest CT, pathologic examination, and RNA sequencing in a rat model
Source: Sci Rep. 2021 Mar 18;11:6318. doi: 10.1038/s41598-021-85662-z (PMC7973781; doi:10.1038/s41598-021-85662-z)
Supplement: Supplementary file 1 — Supplementary Files [file 41598_2021_85662_MOESM1_ESM.docx]

**Evaluation of polyhexamethylene guanidine-induced lung injuries by chest CT, pathologic examination, and RNA sequencing in a rat model**

Cherry Kim^1^, MD, PhD*, Sang Hoon Jeong^2,3^, PhD*, Jaeyoung Kim^2,3^, PhD, Ki Yeol Lee^1^, MD, PhD, Jaehyung Cha^3^, PhD, Chang Hyun Lee^4^, MD, PhD, Eun-Kee Park^5^, PhD, Ju-Han Lee^6^, MD, PhD

*Equally contributed authors

^1^Department of Radiology, Ansan Hospital, Korea University College of Medicine, 123, Jeokgeum-ro, Danwon-gu, Ansan-si, Gyeonggi, 15355, South Korea

^2^Research Institute for Skin Image, Korea University College of Medicine, 123, Jeokgeum-ro, Danwon-gu, Ansan-si, Gyeonggi, 15355, South Korea

^3^Medical Science research center, Ansan Hospital, Korea University College of Medicine, 123, Jeokgeum-ro, Danwon-gu, Ansan-si, Gyeonggi, 15355, South Korea

^4^Department of Radiology, College of Medicine, Seoul National University, Seoul National University Hospital, Seoul, 03080, South Korea

^5^Department of Medical Humanities and Social Medicine, College of Medicine, Kosin University, Busan, 49267, South Korea

^6^Department of Pathology, Ansan Hospital, Korea University College of Medicine, 123, Jeokgeum-ro, Danwon-gu, Ansan-si, Gyeonggi, 15355, South Korea

**Supplementary Figure S1**. The pathologic finding of bronchiolar-alveolar adenoma (H&E, x40). A solid mass of high epithelial cell density is observed (arrowheads). Alveolar spaces are obliterated by proliferating tumor cells.


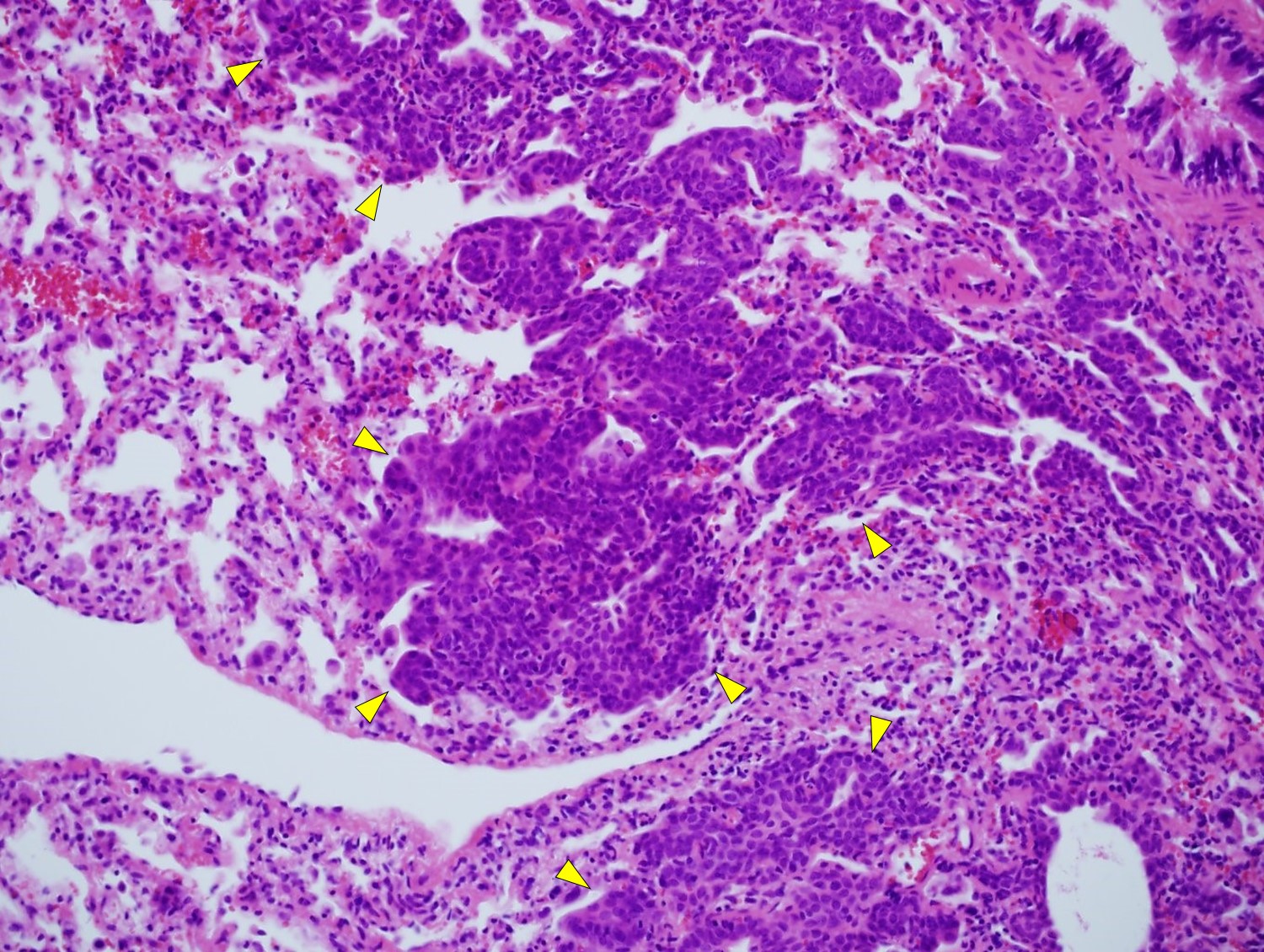


**Supplementary Figure S2**. The extent of inflammation and fibrosis according to the number of weeks of exposure.


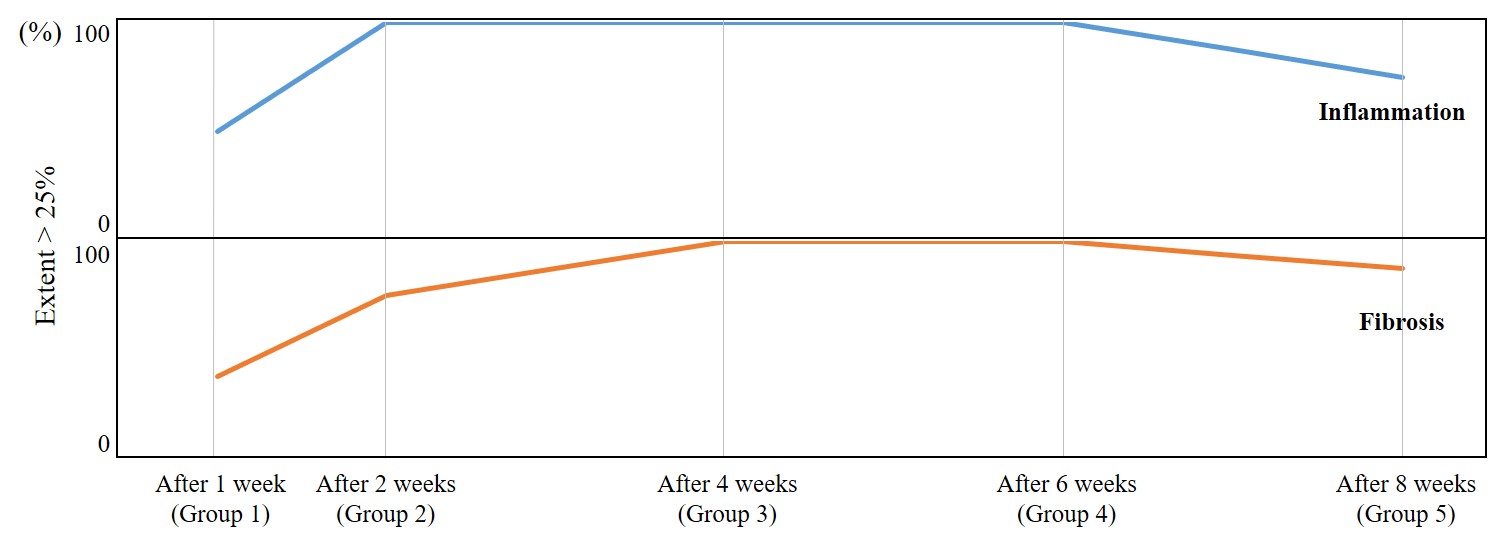


**Supplementary Figure S3.** Analysis of myofibroblast markers in lung tissues. (A) Western blotting results of fibronectin, collagen type I, and α-SMA expression in the lung tissues of rats sacrificed at weeks 4, 6, and 8 weeks after intratracheal instillation of PHMG. (B) Raw data of western blots.


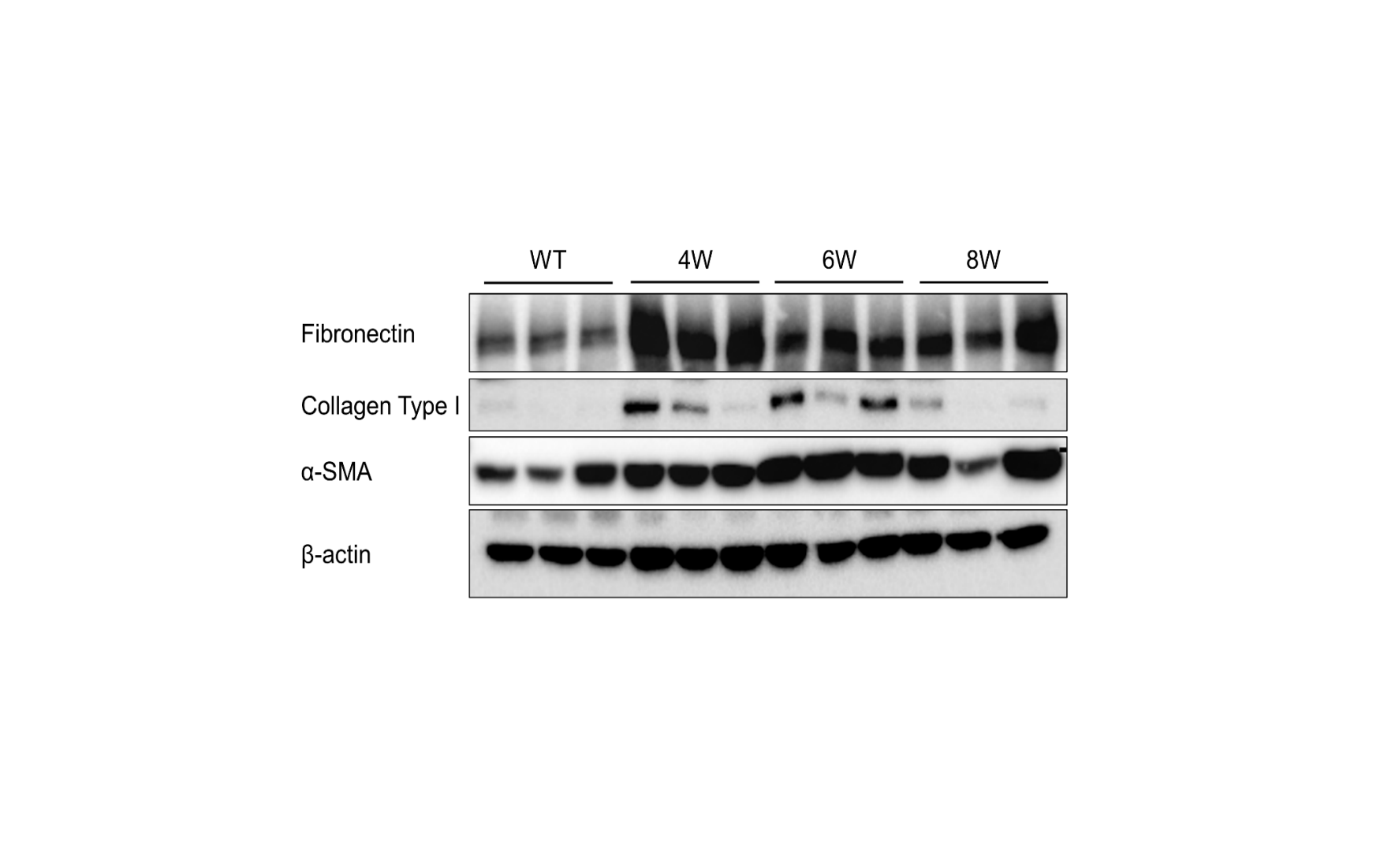

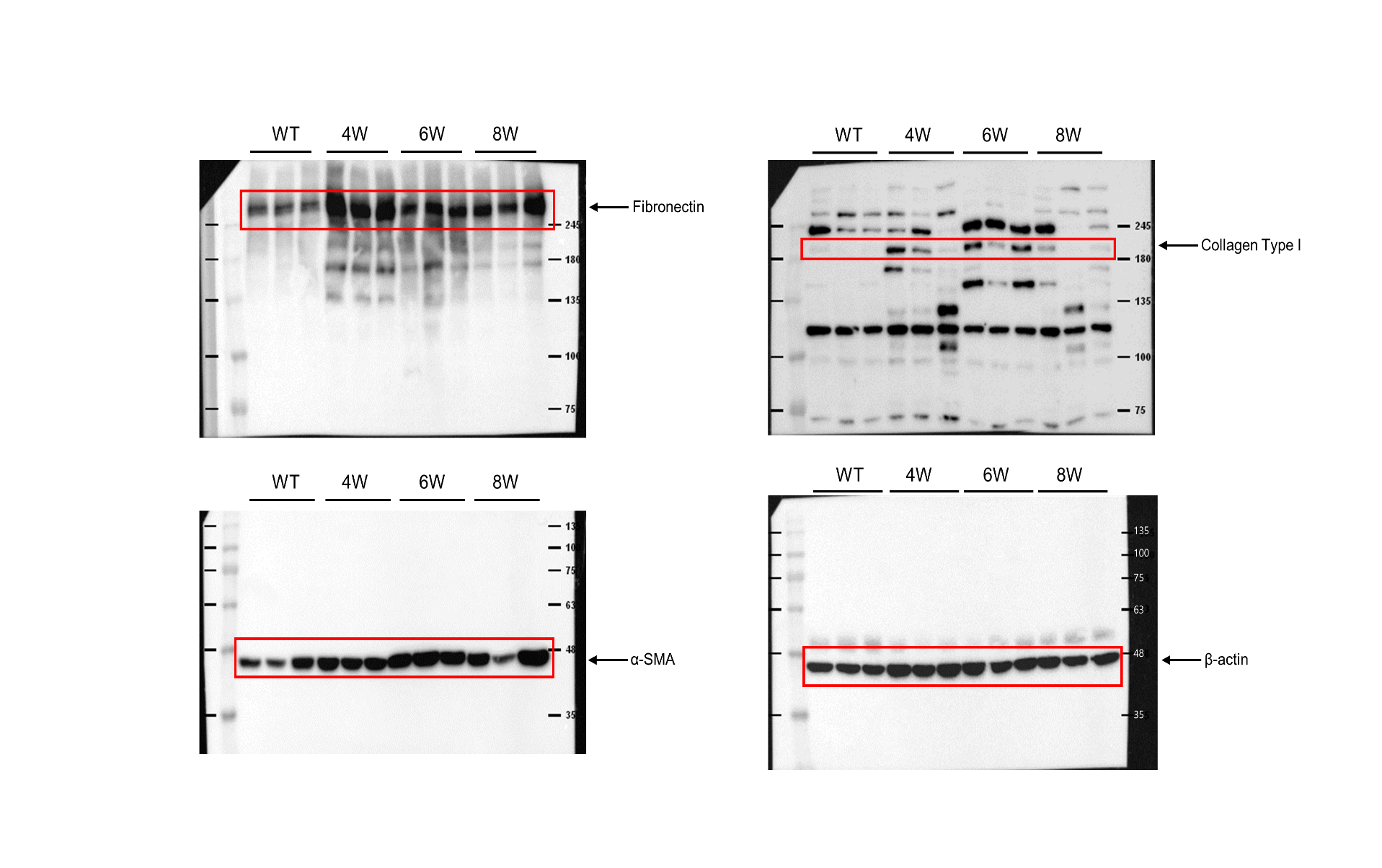


**a**

**b**


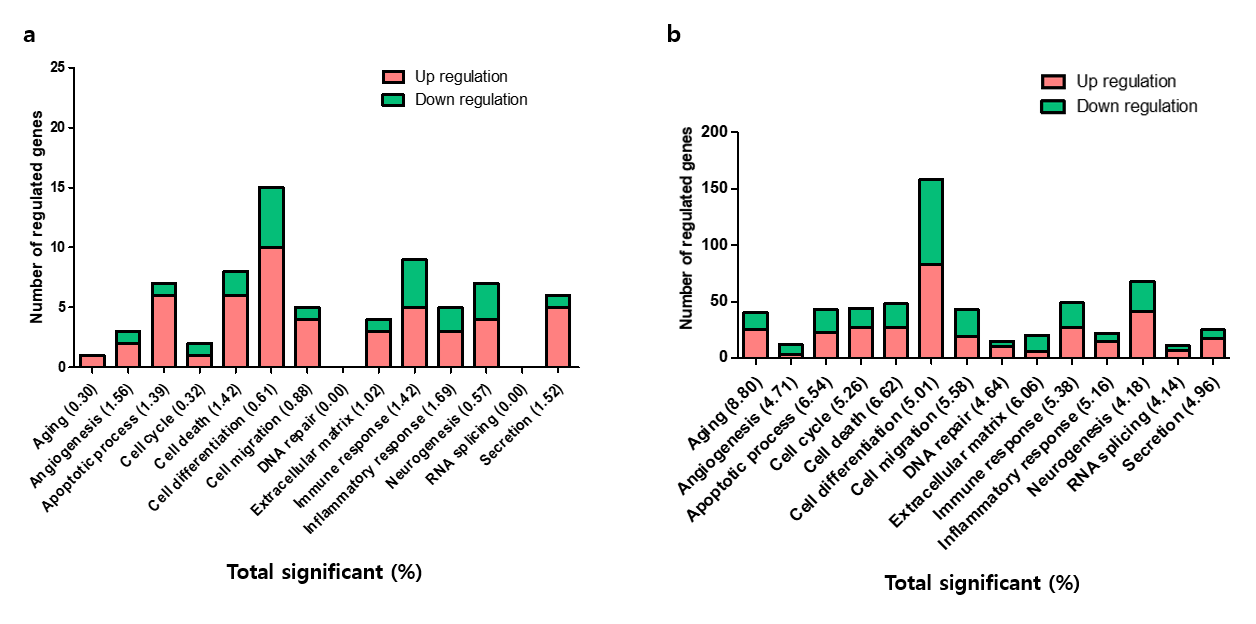
**Supplementary Figure S4**. The number of genes belonging to the 14 randomly selected gene categories using gene ontology (red: upregulated genes, green: downregulated genes). (A) Results at 4 weeks post-PHMG exposure. (B) Results at 8 weeks post-PHMG exposure. The percent value of changed genes among the total genes in each category.

**Supplementary Figure S5**. Photograph of the modified videoscope used for intratracheal instillation.


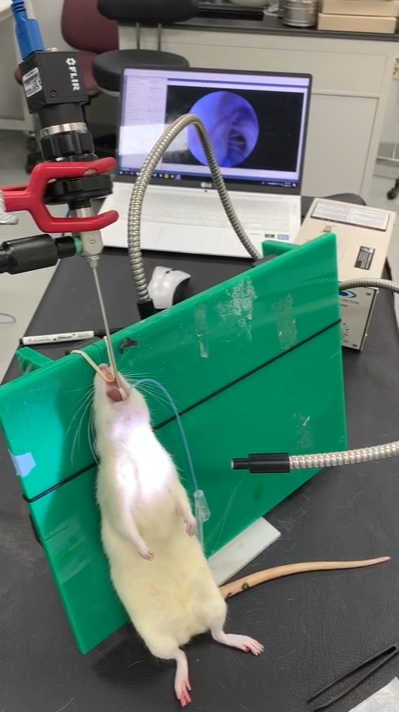


| **Supplementary Table S1**. Major CT findings (peribronchial GGO, centrilobular nodules, diffuse GGO, and linear densities and nodules) and matched major and minor histologic findings according to the groups. | | |
| --- | --- | --- |
| **Major CT finding** | **Matched major histologic findings** | **Minor histologic findings** |
| *After 1 week (Group 1)* | | |
| Peribronchial GGO (n=8) | Infiltrate of histiocytes in peribronchial spaces (n=7) | Focal infiltrate of histiocytes in alveolar spaces (n=5) |
|  | Moderate fibrosis in peribronchial/alveolar spaces (n=1) | Mild fibrotic foci in peribronchial/alveolar spaces (n=2) |
| *After 2 weeks (Group 2)* | | |
| Peribronchial GGO (n=6) | Infiltrate of histiocytes in peribronchial/alveolar spaces (n=6) | Mild fibrotic foci in peribronchial/alveolar spaces (n=4) |
|  |  | Squamous metaplasia of alveoli in peribronchial spaces (n=1) |
| Diffuse GGO (n=1) | Infiltrate of histiocytes in alveolar spaces (n=1) | N/A |
| Centrilobular nodules (n=1) | Infiltrate of histiocytes and lymphocytes in peribronchial/alveolar spaces (n=1) | Mild fibrotic foci in peribronchial/alveolar spaces (n=1) |
| *After 4 weeks (Group 3)* | | |
| Peribronchial GGO (n=5) | Infiltrate of histiocytes and lymphocytes in peribronchial spaces (n=4) | Foamy histiocytes in alveolar spaces (n=2) |
|  | Marked fibrosis in peribronchial spaces (n=1) | Mild fibrosis in peribronchial spaces (n=1) |
| Centrilobular nodules (n=3) | Fibrosis in peribronchial/alveolar spaces (n=2) | Infiltrate of histiocytes and lymphocytes in peribronchial spaces (n=2) |
|  | Infiltrate of histiocytes and lymphocytes forms nodule-like lesion (n=1) |  |
| *After 6 weeks (Group 4)* | | |
| Peribronchial GGO (n=7) | Infiltrate of histiocytes and lymphocytes in peribronchial/alveolar spaces (n=5) | Mucous cell metaplasia and fibrosis in peribronchial space (n=5) |
|  |  | Foamy histiocytes in alveolar spaces (n=1) |
|  | Marked fibrosis in peribronchial/alveolar spaces space (n=2) | Mucous cell metaplasia and infiltrate of histiocytes and lymphocytes (n=1) |
|  |  | Bronchiolo-alveolar adenoma (n=1) |
| Centrilobular nodules (n=1) | Marked fibrosis in peribronchial space (n=1) | Bronchiolo-alveolar adenoma (n=1) |
| *After 8 weeks (Group 5)* | | |
| Linear densities and nodules (n=8) | Infiltrate of histiocytes and lymphocytes in peribronchial/alveolar spaces (n=5) | Infiltrate of histiocytes and lymphocytes in alveolar spaces (n=2) |
|  | Fibrosis in peribronchial/alveolar spaces (n=2) | Bronchiolo-alveolar adenoma (n=2) |
|  | Bronchiolo-alveolar adenomas (n=1) | Mucous cell metaplasia in peribronchial space (n=1) |
| Note.—GGO, ground glass opacity | | |

| **Supplementary Table S2**. Lists of significantly upregulated and downregulated genes in the lung tissue after 4 weeks of PHMG instillation compared to the lung tissue of a wild-type rat. | | | |
| --- | --- | --- | --- |
| **Gene symbol** | **Description** | **Fold change** | **P-value** |
| ***Upregulated genes*** | | | |
| Bpifb1 | BPI fold containing family B, member 1 | 35.639 | 0.021 |
| Thrsp | thyroid hormone responsive | 10.620 | 0.020 |
| Snca | synuclein alpha | 9.488 | 0.004 |
| Kcnk13 | potassium two pore domain channel subfamily K member 13 | 5.606 | 0.005 |
| Adora3 | adenosine A3 receptor | 4.949 | 0.049 |
| Hemgn | Hemogen | 4.823 | 0.014 |
| Acta1 | actin, alpha 1, skeletal muscle | 4.687 | 0.043 |
| Amz1 | archaelysin family metallopeptidase 1 | 4.249 | 0.033 |
| Pde1a | phosphodiesterase 1A | 3.811 | 0.010 |
| Plcd4 | phospholipase C, delta 4 | 3.648 | 0.013 |
| Hmox1 | heme oxygenase 1 | 3.522 | 0.009 |
| Slc24a5 | solute carrier family 24 member 5 | 3.367 | 0.015 |
| Ror2 | receptor tyrosine kinase-like orphan receptor 2 | 3.364 | 0.031 |
| Pdhx | pyruvate dehydrogenase complex, component X | 3.265 | 0.011 |
| Thbs2 | thrombospondin 2 | 3.193 | 0.016 |
| Lcn2 | lipocalin 2 | 3.095 | 0.036 |
| Rnf11l1 | ring finger protein 11-like 1 | 3.066 | 0.002 |
| Chi3l1 | chitinase 3 like 1 | 3.058 | 0.037 |
| Fbxo42 | F-box protein 42 | 2.986 | 0.050 |
| Amtn | Amelotin | 2.960 | 0.017 |
| Trmt11 | tRNA methyltransferase 11 homolog | 2.895 | 0.004 |
| Sgpl1 | sphingosine-1-phosphate lyase 1 | 2.879 | 0.019 |
| Hey2 | hes-related family bHLH transcription factor with YRPW motif 2 | 2.817 | 0.036 |
| Iqgap3 | IQ motif containing GTPase activating protein 3 | 2.815 | 0.042 |
| RragB | Ras-related GTP binding B | 2.770 | 0.001 |
| Vwa7 | von Willebrand factor A domain containing 7 | 2.753 | 0.025 |
| Grb14 | growth factor receptor bound protein 14 | 2.714 | 0.005 |
| Apobr | apolipoprotein B receptor | 2.640 | 0.037 |
| Chac1 | ChaC glutathione-specific gamma-glutamylcyclotransferase 1 | 2.589 | 0.041 |
| Lilrb3 | leukocyte immunoglobulin like receptor B3 | 2.547 | 0.040 |
| Orm1 | orosomucoid 1 | 2.545 | 0.018 |
| Eral1 | Era-like 12S mitochondrial rRNA chaperone 1 | 2.539 | 0.022 |
| Cxcr1 | C-X-C motif chemokine receptor 1 | 2.531 | 0.031 |
| Cep97 | centrosomal protein 97 | 2.464 | 0.041 |
| Sp5 | Sp5 transcription factor | 2.283 | 0.031 |
| Arhgap24 | Rho GTPase activating protein 24 | 2.248 | 0.045 |
| Rbm24 | RNA binding motif protein 24 | 2.245 | 0.039 |
| Specc1 | sperm antigen with calponin homology and coiled-coil domains 1 | 2.238 | 0.020 |
| Rnf128 | ring finger protein 128, E3 ubiquitin protein ligase | 2.221 | 0.045 |
| Gpn3 | GPN-loop GTPase 3 | 2.211 | 0.041 |
| Alox15 | arachidonate 15-lipoxygenase | 2.211 | 0.018 |
| Bco2 | beta-carotene oxygenase 2 | 2.207 | 0.002 |
| Slc2a13 | solute carrier family 2 member 13 | 2.148 | 0.016 |
| Rhd | Rh blood group, D antigen | 2.147 | 0.041 |
| Cnnm2 | cyclin and CBS domain divalent metal cation transport mediator 2 | 2.111 | 0.044 |
| Fcer1a | Fc fragment of IgE receptor Ia | 2.108 | 0.022 |
| Rab11fip2 | RAB11 family interacting protein 2 | 2.107 | 0.045 |
| Megf9 | multiple EGF-like-domains 9 | 2.102 | 0.009 |
| Tyro3 | TYRO3 protein tyrosine kinase | 2.100 | 0.006 |
| Gcat | glycine C-acetyltransferase | 2.095 | 0.019 |
| Lhfpl2 | lipoma HMGIC fusion partner-like 2 | 2.083 | 0.012 |
| Creg1 | cellular repressor of E1A-stimulated genes 1 | 2.073 | 0.034 |
| Serpina10 | serpin peptidase inhibitor, clade A (alpha-1 antiproteinase, antitrypsin), member 10 | 2.073 | 0.049 |
| Sirt4 | sirtuin 4 | 2.057 | 0.021 |
| Stxbp5 | syntaxin binding protein 5 | 2.041 | 0.020 |
| Dmbt1 | deleted in malignant brain tumors 1 | 2.033 | 0.014 |
| Asf1b | anti-silencing function 1B histone chaperone | 2.007 | 0.024 |
| ***Downregulated genes*** | | | |
| Tmem255b | transmembrane protein 255B | 0.499 | 0.006 |
| Cox6b2 | cytochrome c oxidase subunit VIb polypeptide 2 | 0.499 | 0.005 |
| RGD1564482 | RGD1564482 | 0.466 | 0.024 |
| Cd8a | CD8a molecule | 0.465 | 0.006 |
| Sh2d2a | SH2 domain containing 2A | 0.461 | 0.024 |
| Kif5c | kinesin family member 5C | 0.458 | 0.037 |
| Lax1 | lymphocyte transmembrane adaptor 1 | 0.454 | 0.026 |
| Nrg4 | neuregulin 4 | 0.447 | 0.046 |
| Ephb1 | Eph receptor B1 | 0.441 | 0.034 |
| Rbp4 | retinol binding protein 4 | 0.433 | 0.008 |
| Gzmm | granzyme M | 0.418 | 0.047 |
| Sncaip | synuclein, alpha interacting protein | 0.412 | 0.039 |
| Spry2 | sprouty RTK signaling antagonist 2 | 0.412 | 0.018 |
| Cmc1 | C-x(9)-C motif containing 1 | 0.395 | 0.021 |
| Rspo1 | R-spondin 1 | 0.395 | 0.048 |
| Gata4 | GATA binding protein 4 | 0.385 | 0.022 |
| Bmp7 | bone morphogenetic protein 7 | 0.384 | 0.026 |
| Ptgds | prostaglandin D2 synthase | 0.383 | 0.026 |
| Gle1 | GLE1 RNA export mediator | 0.372 | 0.038 |
| Adh6a | alcohol dehydrogenase 6A (class V) | 0.372 | 0.007 |
| Sit1 | signaling threshold regulating transmembrane adaptor 1 | 0.354 | 0.021 |
| Art2b | ADP-ribosyltransferase 2b | 0.345 | 0.019 |
| Wnt2b | wingless-type MMTV integration site family, member 2B | 0.341 | 0.015 |
| Map3k8 | mitogen-activated protein kinase kinase kinase 8 | 0.338 | 0.044 |
| Grap2 | GRB2-related adaptor protein 2 | 0.331 | 0.040 |
| Acot1 | acyl-CoA thioesterase 1 | 0.305 | 0.024 |
| Rnase17 | ribonuclease 17 | 0.289 | 0.032 |
| Tnfrsf25 | TNF receptor superfamily member 25 | 0.245 | 0.026 |
| Calml3 | calmodulin-like 3 | 0.214 | 0.018 |
| Pou2f3 | POU class 2 homeobox 3 | 0.214 | 0.050 |
| Ear11 | eosinophil-associated, ribonuclease A family, member 11 | 0.199 | 0.007 |
| Ces1c | carboxylesterase 1C | 0.145 | 0.009 |

| **Supplementary Table S3.** Lists of significantly upregulated and downregulated genes in the lung tissue after 8 weeks of PHMG instillation compared to the lung tissue of a wild-type rat. | | | |
| --- | --- | --- | --- |
| **Gene symbol** | **Description** | **Fold change** | **P-value** |
| ***Upregulated genes*** | | | |
| Bpifa1 | BPI fold containing family A, member 1 | 45.195 | 0.043 |
| Rnaseh1 | ribonuclease H1 | 34.647 | 0.011 |
| Rn5-8s | 5.8S ribosomal RNA | 27.895 | 0.006 |
| Pdha2 | pyruvate dehydrogenase E1 alpha 2 | 19.673 | 0.029 |
| Ccpg1os | cell cycle progression 1, opposite strand | 19.364 | 0.005 |
| Slpil2 | antileukoproteinase-like 2 | 19.000 | 0.001 |
| Fam71e2 | family with sequence similarity 71, member E2 | 16.751 | 0.030 |
| Smtnl1 | smoothelin-like 1 | 13.492 | 0.002 |
| Serpinb11 | serpin peptidase inhibitor, clade B (ovalbumin), member 11 | 13.391 | 0.050 |
| Acpp | acid phosphatase, prostate | 13.355 | 0.040 |
| Fam196a | family with sequence similarity 196, member A | 13.083 | 0.010 |
| Cinp | cyclin-dependent kinase 2-interacting protein | 12.914 | 0.026 |
| Klf2 | Kruppel-like factor 2 | 12.655 | 0.009 |
| Mpc2 | mitochondrial pyruvate carrier 2 | 12.158 | 0.017 |
| Cep76 | centrosomal protein 76 | 12.140 | 0.023 |
| A2m | alpha-2-macroglobulin | 12.108 | 0.005 |
| Hdhd2 | haloacid dehalogenase-like hydrolase domain containing 2 | 11.524 | 0.000 |
| Snca | synuclein alpha | 10.625 | 0.007 |
| Mfsd7 | major facilitator superfamily domain containing 7 | 10.289 | 0.016 |
| Idh1 | isocitrate dehydrogenase (NADP(+)) 1, cytosolic | 9.811 | 0.017 |
| Ppp4c | protein phosphatase 4, catalytic subunit | 9.561 | 0.003 |
| Klra1 | killer cell lectin-like receptor, subfamily A, member 1 | 9.488 | 0.006 |
| Gpd2 | glycerol-3-phosphate dehydrogenase 2 | 9.229 | 0.020 |
| LOC500594 | LRRGT00162 | 9.195 | 0.002 |
| Spdl1 | spindle apparatus coiled-coil protein 1 | 9.047 | 0.033 |
| Dusp5 | dual specificity phosphatase 5 | 8.917 | 0.025 |
| LOC498231 | LRRGT00144 | 8.879 | 0.006 |
| Seli | selenoprotein I | 8.744 | 0.011 |
| Rasl2-9 | RAS-like, family 2, locus 9 | 8.693 | 0.012 |
| Thap1 | THAP domain containing 1 | 8.675 | 0.002 |
| Prtg | protogenin | 8.654 | 0.002 |
| Pdia4 | protein disulfide isomerase family A, member 4 | 8.551 | 0.034 |
| Fads3 | fatty acid desaturase 3 | 8.146 | 0.000 |
| Gimap1 | GTPase, IMAP family member 1 | 7.977 | 0.020 |
| Adora3 | adenosine A3 receptor | 7.937 | 0.007 |
| LOC100361645 | LRRGT00075-like | 7.899 | 0.032 |
| Ctns | cystinosin, lysosomal cystine transporter | 7.433 | 0.000 |
| Pparg | peroxisome proliferator-activated receptor gamma | 7.342 | 0.004 |
| Fmo3 | flavin containing monooxygenase 3 | 7.164 | 0.004 |
| LOC360933 | similar to Ac1591 | 7.070 | 0.005 |
| Harbi1 | harbinger transposase derived 1 | 7.065 | 0.048 |
| Stxbp3 | syntaxin binding protein 3 | 6.936 | 0.017 |
| LOC100363289 | LRRGT00022-like | 6.901 | 0.032 |
| Ccdc65 | coiled-coil domain containing 65 | 6.748 | 0.049 |
| Spcs3 | signal peptidase complex subunit 3 | 6.636 | 0.019 |
| Mdga1 | MAM domain containing glycosylphosphatidylinositol anchor 1 | 6.616 | 0.046 |
| Scd1 |  | 6.436 | 0.002 |
| Xpa | XPA, DNA damage recognition and repair factor | 6.178 | 0.045 |
| Nup107 | nucleoporin 107 | 6.153 | 0.001 |
| Apaf1 | apoptotic peptidase activating factor 1 | 6.139 | 0.020 |
| Tlr11 | toll-like receptor 11 | 6.132 | 0.007 |
| Slc7a11 | solute carrier family 7 member 11 | 6.055 | 0.000 |
| Zfp281 | zinc finger protein 281 | 6.008 | 0.005 |
| Atn1 | atrophin 1 | 5.986 | 0.007 |
| Sfxn2 | sideroflexin 2 | 5.976 | 0.008 |
| Rnf5 | ring finger protein 5, E3 ubiquitin protein ligase | 5.962 | 0.026 |
| Gsr | glutathione-disulfide reductase | 5.925 | 0.025 |
| Xcr1 | X-C motif chemokine receptor 1 | 5.908 | 0.048 |
| Lpl | lipoprotein lipase | 5.839 | 0.047 |
| Adar | adenosine deaminase, RNA-specific | 5.748 | 0.017 |
| Gtse1 | G-2 and S-phase expressed 1 | 5.662 | 0.002 |
| Bbs5 | Bardet-Biedl syndrome 5 | 5.626 | 0.017 |
| Tfec | transcription factor EC | 5.622 | 0.032 |
| Ston2 | stonin 2 | 5.537 | 0.018 |
| Btbd9 | BTB domain containing 9 | 5.531 | 0.018 |
| Pias1 | protein inhibitor of activated STAT, 1 | 5.471 | 0.001 |
| Atn1 | atrophin 1 | 5.431 | 0.008 |
| Pex13 | peroxisomal biogenesis factor 13 | 5.409 | 0.032 |
| Cndp1 | carnosine dipeptidase 1 | 5.354 | 0.036 |
| Fcgr2a | Fc fragment of IgG, low affinity IIa, receptor | 5.352 | 0.022 |
| Erich1 | glutamate-rich 1 | 5.342 | 0.018 |
| Dusp10 | dual specificity phosphatase 10 | 5.305 | 0.001 |
| Trnau1ap | tRNA selenocysteine 1 associated protein 1 | 5.302 | 0.043 |
| Faim | Fas apoptotic inhibitory molecule | 5.297 | 0.008 |
| Mettl25 | methyltransferase like 25 | 5.181 | 0.040 |
| Casc5 |  | 5.160 | 0.011 |
| Cybb | cytochrome b-245 beta chain | 5.156 | 0.026 |
| Top2a | topoisomerase (DNA) II alpha | 5.102 | 0.003 |
| Sipa1 | signal-induced proliferation-associated 1 | 5.093 | 0.032 |
| Gtpbp10 | GTP binding protein 10 | 5.042 | 0.039 |
| Slc9a3r1 | SLC9A3 regulator 1 | 5.031 | 0.002 |
| Dbt | dihydrolipoamide branched chain transacylase E2 | 4.843 | 0.019 |
| Stx17 | syntaxin 17 | 4.690 | 0.014 |
| Xrcc4 | X-ray repair cross complementing 4 | 4.668 | 0.021 |
| Slc25a5 | solute carrier family 25 member 5 | 4.638 | 0.000 |
| Casp4 | caspase 4 | 4.637 | 0.047 |
| Bsdc1 | BSD domain containing 1 | 4.631 | 0.045 |
| Mdga2 | MAM domain containing glycosylphosphatidylinositol anchor 2 | 4.501 | 0.039 |
| Gid8 | GID complex subunit 8 | 4.496 | 0.018 |
| Tmem101 | transmembrane protein 101 | 4.470 | 0.018 |
| LOC306079 | similar to RIKEN cDNA 3100001N19 | 4.434 | 0.011 |
| Trappc2b | trafficking protein particle complex 2B | 4.417 | 0.026 |
| Trim24 | tripartite motif-containing 24 | 4.270 | 0.028 |
| Tex26 | testis expressed 26 | 4.257 | 0.043 |
| Pwp2 | PWP2 periodic tryptophan protein homolog (yeast) | 4.254 | 0.011 |
| Ost4 | oligosaccharyltransferase complex subunit 4 | 4.233 | 0.022 |
| Bub1b | BUB1 mitotic checkpoint serine/threonine kinase B | 4.207 | 0.003 |
| Ccno | cyclin O | 4.198 | 0.041 |
| Pfn1 | profilin 1 | 4.192 | 0.006 |
| Kif9 | kinesin family member 9 | 4.115 | 0.002 |
| Lrrc71 | leucine rich repeat containing 71 | 4.099 | 0.000 |
| Rnf11l1 | ring finger protein 11-like 1 | 4.063 | 0.002 |
| Vldlr | very low density lipoprotein receptor | 4.045 | 0.003 |
| Tex12 | testis expressed 12 | 4.031 | 0.044 |
| Mylip | myosin regulatory light chain interacting protein | 4.020 | 0.036 |
| Clec4a3 | C-type lectin domain family 4, member A3 | 4.009 | 0.007 |
| Hexim2 | hexamethylene bis-acetamide inducible 2 | 3.869 | 0.013 |
| Gphn | gephyrin | 3.853 | 0.026 |
| Jak2 | Janus kinase 2 | 3.850 | 0.003 |
| Ak3 | adenylate kinase 3 | 3.827 | 0.000 |
| Shmt1 | serine hydroxymethyltransferase 1 | 3.806 | 0.041 |
| Chdh | choline dehydrogenase | 3.764 | 0.015 |
| Myc | myelocytomatosis oncogene | 3.757 | 0.003 |
| LOC314140 | ribose-phosphate pyrophosphokinase I -like | 3.747 | 0.020 |
| Wnt3a | wingless-type MMTV integration site family, member 3A | 3.722 | 0.010 |
| Trmt11 | tRNA methyltransferase 11 homolog | 3.720 | 0.003 |
| Ssbp1 | single stranded DNA binding protein 1 | 3.719 | 0.014 |
| Dclre1a | DNA cross-link repair 1A | 3.666 | 0.018 |
| H2afx | H2A histone family, member X | 3.657 | 0.014 |
| Slco4a1 | solute carrier organic anion transporter family, member 4a1 | 3.648 | 0.010 |
| Osbpl11 | oxysterol binding protein-like 11 | 3.645 | 0.016 |
| Gstm2 | glutathione S-transferase mu 2 | 3.639 | 0.024 |
| Sbds | SBDS ribosome assembly guanine nucleotide exchange factor | 3.613 | 0.047 |
| Ankrd42 | ankyrin repeat domain 42 | 3.539 | 0.011 |
| Psmd10 | proteasome 26S subunit, non-ATPase 10 | 3.537 | 0.012 |
| Pbx3 | PBX homeobox 3 | 3.531 | 0.038 |
| Ubqln2 | ubiquilin 2 | 3.526 | 0.012 |
| Mad2l1 | MAD2 mitotic arrest deficient-like 1 (yeast) | 3.518 | 0.026 |
| Tefm | transcription elongation factor, mitochondrial | 3.517 | 0.013 |
| Slc30a7 | solute carrier family 30 member 7 | 3.507 | 0.042 |
| Dhx32 | DEAH-box helicase 32 (putative) | 3.476 | 0.011 |
| Mapk9 | mitogen-activated protein kinase 9 | 3.461 | 0.015 |
| Cox7c | cytochrome c oxidase subunit 7C | 3.457 | 0.001 |
| Phykpl | 5-phosphohydroxy-L-lysine phospho-lyase | 3.453 | 0.025 |
| Senp3 | Sumo1/sentrin/SMT3 specific peptidase 3 | 3.446 | 0.044 |
| Dock7 | dedicator of cytokinesis 7 | 3.421 | 0.006 |
| Exoc6 | exocyst complex component 6 | 3.420 | 0.000 |
| Ensa | endosulfine alpha | 3.414 | 0.010 |
| Kif11 | kinesin family member 11 | 3.412 | 0.020 |
| Msh2 | mutS homolog 2 | 3.405 | 0.007 |
| Milr1 | mast cell immunoglobulin-like receptor 1 | 3.386 | 0.034 |
| LOC500684 | hypothetical protein LOC500684 | 3.384 | 0.031 |
| Mterfd1 |  | 3.376 | 0.030 |
| Fam55b |  | 3.370 | 0.007 |
| Tdrd7 | tudor domain containing 7 | 3.365 | 0.047 |
| Rnf182 | ring finger protein 182 | 3.364 | 0.034 |
| Ces1e |  | 3.364 | 0.038 |
| Tial1 | Tia1 cytotoxic granule-associated RNA binding protein-like 1 | 3.364 | 0.030 |
| Hspa9 | heat shock protein family A member 9 | 3.359 | 0.010 |
| Snrpf | small nuclear ribonucleoprotein polypeptide F | 3.352 | 0.003 |
| Lrp12 | LDL receptor related protein 12 | 3.337 | 0.012 |
| Zc3h6 | zinc finger CCCH type containing 6 | 3.336 | 0.036 |
| Tmed7 | transmembrane p24 trafficking protein 7 | 3.333 | 0.004 |
| Arr3 | arrestin 3, retinal (X-arrestin) | 3.286 | 0.048 |
| Csgalnact1 | chondroitin sulfate N-acetylgalactosaminyltransferase 1 | 3.242 | 0.028 |
| Gpr160 | G protein-coupled receptor 160 | 3.233 | 0.006 |
| Plk2 | polo-like kinase 2 | 3.208 | 0.010 |
| Slc18a2 | solute carrier family 18 member A2 | 3.199 | 0.011 |
| Orc3 | origin recognition complex, subunit 3 | 3.193 | 0.038 |
| Nos3 | nitric oxide synthase 3 | 3.165 | 0.046 |
| Tnfrsf4 | TNF receptor superfamily member 4 | 3.162 | 0.008 |
| Polr3g | polymerase (RNA) III subunit G | 3.142 | 0.027 |
| Commd1 | copper metabolism domain containing 1 | 3.138 | 0.010 |
| Sft2d1 | SFT2 domain containing 1 | 3.123 | 0.027 |
| Ttc30b | tetratricopeptide repeat domain 30B | 3.120 | 0.003 |
| Cenpu | centromere protein U | 3.099 | 0.017 |
| Il6r | interleukin 6 receptor | 3.067 | 0.000 |
| RGD1565685 | similar to RIKEN cDNA 1810030O07 | 3.033 | 0.001 |
| Ccr5 | chemokine (C-C motif) receptor 5 | 3.012 | 0.027 |
| Atp5s | ATP synthase, H+ transporting, mitochondrial Fo complex, subunit s (factor B) | 3.007 | 0.039 |
| Pomt2 | protein-O-mannosyltransferase 2 | 3.002 | 0.039 |
| Prkx | protein kinase, X-linked | 2.978 | 0.019 |
| Pitrm1 | pitrilysin metallopeptidase 1 | 2.963 | 0.030 |
| Ankrd13c | ankyrin repeat domain 13C | 2.958 | 0.001 |
| Cstb | cystatin B | 2.954 | 0.020 |
| Ostm1 | osteopetrosis associated transmembrane protein 1 | 2.954 | 0.010 |
| Npl | N-acetylneuraminate pyruvate lyase | 2.925 | 0.025 |
| Tpbg | trophoblast glycoprotein | 2.918 | 0.009 |
| Mrc1 | mannose receptor, C type 1 | 2.917 | 0.011 |
| Rab27a | RAB27A, member RAS oncogene family | 2.915 | 0.014 |
| Irf9 | interferon regulatory factor 9 | 2.914 | 0.005 |
| Drc7 | dynein regulatory complex subunit 7 | 2.894 | 0.042 |
| Rpo1-3 |  | 2.892 | 0.024 |
| Sephs2 | selenophosphate synthetase 2 | 2.867 | 0.018 |
| Chst1 | carbohydrate sulfotransferase 1 | 2.864 | 0.004 |
| Hiat1 |  | 2.860 | 0.037 |
| Pde4dip | phosphodiesterase 4D interacting protein | 2.859 | 0.011 |
| Sectm1a | secreted and transmembrane 1A | 2.851 | 0.002 |
| Tada1 | transcriptional adaptor 1 | 2.847 | 0.047 |
| Sptssa | serine palmitoyltransferase, small subunit A | 2.836 | 0.018 |
| Foxj2 | forkhead box J2 | 2.835 | 0.046 |
| Wdhd1 | WD repeat and HMG-box DNA binding protein 1 | 2.834 | 0.025 |
| RGD1308706 | similar to RIKEN cDNA 4921524J17 | 2.831 | 0.014 |
| Fgd4 | FYVE, RhoGEF and PH domain containing 4 | 2.831 | 0.023 |
| Tbc1d31 | TBC1 domain family, member 31 | 2.821 | 0.018 |
| Slc20a2 | solute carrier family 20 member 2 | 2.813 | 0.039 |
| Son | Son DNA binding protein | 2.811 | 0.010 |
| Ccdc47 | coiled-coil domain containing 47 | 2.802 | 0.007 |
| Dnah1 | dynein, axonemal, heavy chain 1 | 2.802 | 0.032 |
| Runx2 | runt-related transcription factor 2 | 2.801 | 0.005 |
| Tmem5 | transmembrane protein 5 | 2.798 | 0.050 |
| Epha7 | Eph receptor A7 | 2.793 | 0.040 |
| Prkca | protein kinase C, alpha | 2.788 | 0.042 |
| Cited2 | Cbp/p300-interacting transactivator, with Glu/Asp-rich carboxy-terminal domain, 2 | 2.787 | 0.003 |
| Sec62 | SEC62 homolog, preprotein translocation factor | 2.781 | 0.000 |
| Depdc7 | DEP domain containing 7 | 2.763 | 0.019 |
| Ccdc34 | coiled-coil domain containing 34 | 2.750 | 0.003 |
| Dnmt3a | DNA methyltransferase 3 alpha | 2.741 | 0.007 |
| Naaa | N-acylethanolamine acid amidase | 2.737 | 0.050 |
| Rasd1 | ras related dexamethasone induced 1 | 2.727 | 0.032 |
| Sbf2 | SET binding factor 2 | 2.720 | 0.003 |
| Kcnc3 | potassium voltage-gated channel subfamily C member 3 | 2.720 | 0.007 |
| Dpp9 | dipeptidyl peptidase 9 | 2.710 | 0.006 |
| Herc3 | HECT and RLD domain containing E3 ubiquitin protein ligase 3 | 2.687 | 0.000 |
| Cpped1 | calcineurin-like phosphoesterase domain containing 1 | 2.665 | 0.018 |
| Abcc1 | ATP binding cassette subfamily C member 1 | 2.656 | 0.024 |
| Pts | 6-pyruvoyl-tetrahydropterin synthase | 2.641 | 0.020 |
| Naa40 | N(alpha)-acetyltransferase 40, NatD catalytic subunit | 2.638 | 0.040 |
| Adap2 | ArfGAP with dual PH domains 2 | 2.632 | 0.020 |
| Raf1 | Raf-1 proto-oncogene, serine/threonine kinase | 2.627 | 0.025 |
| Anxa1 | annexin A1 | 2.626 | 0.046 |
| Qsox2 | quiescin sulfhydryl oxidase 2 | 2.613 | 0.003 |
| Clec7a | C-type lectin domain family 7, member A | 2.612 | 0.002 |
| Baz1a | bromodomain adjacent to zinc finger domain, 1A | 2.610 | 0.010 |
| Pex3 | peroxisomal biogenesis factor 3 | 2.604 | 0.018 |
| Slc35f6 | solute carrier family 35, member F6 | 2.601 | 0.046 |
| P2ry2 | purinergic receptor P2Y2 | 2.574 | 0.025 |
| RGD1565059 | similar to hypothetical protein E130311K13 | 2.571 | 0.039 |
| Tpp1 | tripeptidyl peptidase 1 | 2.570 | 0.007 |
| Erlin1 | ER lipid raft associated 1 | 2.562 | 0.014 |
| Impact | impact RWD domain protein | 2.560 | 0.001 |
| Hist1h2bh | histone cluster 1, H2bh | 2.559 | 0.049 |
| Rad50 | RAD50 double strand break repair protein | 2.557 | 0.046 |
| Gla | galactosidase, alpha | 2.554 | 0.023 |
| Bfar | bifunctional apoptosis regulator | 2.553 | 0.008 |
| Tbc1d9 | TBC1 domain family member 9 | 2.548 | 0.025 |
| Cenpw | centromere protein W | 2.546 | 0.042 |
| Eif2s1 | eukaryotic translation initiation factor 2 subunit 1 alpha | 2.545 | 0.020 |
| Hopx | HOP homeobox | 2.538 | 0.048 |
| Hspe1 | heat shock protein family E member 1 | 2.531 | 0.008 |
| Fem1b | fem-1 homolog B | 2.530 | 0.022 |
| F2 | coagulation factor II | 2.529 | 0.004 |
| Guf1 | GUF1 homolog, GTPase | 2.511 | 0.024 |
| Tcea1 | transcription elongation factor A (SII) 1 | 2.510 | 0.049 |
| Ddx28 | DEAD-box helicase 28 | 2.504 | 0.025 |
| Lingo4 | leucine rich repeat and Ig domain containing 4 | 2.499 | 0.040 |
| Pip5k1b | phosphatidylinositol-4-phosphate 5-kinase type 1 beta | 2.493 | 0.026 |
| Eif4g3 | eukaryotic translation initiation factor 4 gamma, 3 | 2.493 | 0.045 |
| Clec9a | C-type lectin domain family 9, member A | 2.493 | 0.044 |
| Myo1e | myosin IE | 2.492 | 0.037 |
| Tob1 | transducer of ErbB-2.1 | 2.485 | 0.024 |
| Pon1 | paraoxonase 1 | 2.481 | 0.006 |
| Cnksr3 | Cnksr family member 3 | 2.475 | 0.015 |
| Evi2b | ecotropic viral integration site 2B | 2.472 | 0.018 |
| Cyp39a1 | cytochrome P450, family 39, subfamily a, polypeptide 1 | 2.467 | 0.015 |
| Lrrk2 | leucine-rich repeat kinase 2 | 2.467 | 0.007 |
| Itgae | integrin subunit alpha E | 2.458 | 0.003 |
| Dmtn | dematin actin binding protein | 2.449 | 0.008 |
| Eva1a | eva-1 homolog A, regulator of programmed cell death | 2.448 | 0.018 |
| Atg13 | autophagy related 13 | 2.443 | 0.008 |
| Gosr1 | golgi SNAP receptor complex member 1 | 2.441 | 0.006 |
| Mk1 | Mk1 protein | 2.437 | 0.049 |
| Qser1 | glutamine and serine rich 1 | 2.436 | 0.015 |
| Vof16 | ischemia related factor vof-16 | 2.429 | 0.027 |
| Exd2 | exonuclease 3'-5' domain containing 2 | 2.429 | 0.002 |
| Scd2 | stearoyl-Coenzyme A desaturase 2 | 2.427 | 0.008 |
| Idua | iduronidase, alpha-L- | 2.415 | 0.044 |
| Zfp143 | zinc finger protein 143 | 2.404 | 0.004 |
| Ndfip2 | Nedd4 family interacting protein 2 | 2.395 | 0.008 |
| Arid5b | AT-rich interaction domain 5B | 2.393 | 0.005 |
| Arl6ip1 | ADP-ribosylation factor like GTPase 6 interacting protein 1 | 2.378 | 0.028 |
| Peg3 | paternally expressed 3 | 2.374 | 0.005 |
| Fbxl14 | F-box and leucine-rich repeat protein 14 | 2.370 | 0.027 |
| Rtbdn | retbindin | 2.367 | 0.005 |
| Ndufs1 | NADH dehydrogenase (ubiquinone) Fe-S protein 1 | 2.362 | 0.005 |
| Rsbn1l | round spermatid basic protein 1-like | 2.359 | 0.001 |
| Col4a3bp | collagen type IV alpha 3 binding protein | 2.355 | 0.017 |
| Foxp2 | forkhead box P2 | 2.343 | 0.031 |
| Ciao1 | cytosolic iron-sulfur assembly component 1 | 2.333 | 0.005 |
| Mob1a | MOB kinase activator 1A | 2.328 | 0.023 |
| Hn1l | hematological and neurological expressed 1-like | 2.326 | 0.035 |
| Procr | protein C receptor | 2.315 | 0.023 |
| Scyl2 | SCY1 like pseudokinase 2 | 2.310 | 0.046 |
| Cenpf | centromere protein F | 2.306 | 0.046 |
| Dse | dermatan sulfate epimerase | 2.304 | 0.046 |
| Upf2 | UPF2 regulator of nonsense transcripts homolog (yeast) | 2.304 | 0.002 |
| Abcg1 | ATP binding cassette subfamily G member 1 | 2.302 | 0.035 |
| Rsu1 | Ras suppressor protein 1 | 2.301 | 0.020 |
| Vamp8 | vesicle-associated membrane protein 8 | 2.299 | 0.004 |
| Fem1c | fem-1 homolog C | 2.292 | 0.002 |
| Ubl3 | ubiquitin-like 3 | 2.290 | 0.005 |
| Atad2b | ATPase family, AAA domain containing 2B | 2.288 | 0.039 |
| Lman1 | lectin, mannose-binding, 1 | 2.284 | 0.033 |
| Srsf4 | serine and arginine rich splicing factor 4 | 2.274 | 0.019 |
| Ccdc82 | coiled-coil domain containing 82 | 2.268 | 0.007 |
| Tax1bp1 | Tax1 binding protein 1 | 2.261 | 0.013 |
| Gnpnat1 | glucosamine-phosphate N-acetyltransferase 1 | 2.260 | 0.014 |
| Phf1 | PHD finger protein 1 | 2.258 | 0.018 |
| Zfp410 | zinc finger protein 410 | 2.254 | 0.034 |
| M6pr | mannose-6-phosphate receptor, cation dependent | 2.254 | 0.048 |
| Gtf3c5 | general transcription factor IIIC subunit 5 | 2.253 | 0.016 |
| Ddx52 | DEAD-box helicase 52 | 2.248 | 0.009 |
| Man2a1 | mannosidase, alpha, class 2A, member 1 | 2.243 | 0.042 |
| Sec61a2 | Sec61 translocon alpha 2 subunit | 2.241 | 0.007 |
| Hist2h4 | histone cluster 2, H4 | 2.231 | 0.032 |
| Eif3j | eukaryotic translation initiation factor 3, subunit J | 2.231 | 0.014 |
| Cyyr1 | cysteine and tyrosine rich 1 | 2.230 | 0.009 |
| Tmem243 | transmembrane protein 243 | 2.229 | 0.003 |
| Lrp2 | LDL receptor related protein 2 | 2.225 | 0.036 |
| Mettl2b | methyltransferase like 2B | 2.224 | 0.022 |
| Ildr1 | immunoglobulin-like domain containing receptor 1 | 2.214 | 0.046 |
| Phf14 | PHD finger protein 14 | 2.213 | 0.013 |
| Pcmtd1 | protein-L-isoaspartate (D-aspartate) O-methyltransferase domain containing 1 | 2.207 | 0.008 |
| Nck1 | NCK adaptor protein 1 | 2.206 | 0.008 |
| ST7 | suppression of tumorigenicity 7 | 2.202 | 0.038 |
| Etfdh | electron transfer flavoprotein dehydrogenase | 2.200 | 0.025 |
| Vim | vimentin | 2.195 | 0.050 |
| Hsp90b1 | heat shock protein 90 beta family member 1 | 2.195 | 0.017 |
| Brms1l | breast cancer metastasis-suppressor 1-like | 2.190 | 0.037 |
| Pnpt1 | polyribonucleotide nucleotidyltransferase 1 | 2.188 | 0.045 |
| Tceb2 | transcription elongation factor B subunit 2 | 2.185 | 0.040 |
| Cyp51 | cytochrome P450, family 51 | 2.185 | 0.012 |
| Ralgapa1 | Ral GTPase activating protein catalytic alpha subunit 1 | 2.179 | 0.000 |
| Osbpl8 | oxysterol binding protein-like 8 | 2.176 | 0.016 |
| Zfp652 | zinc finger protein 652 | 2.172 | 0.038 |
| LOC680254 | hypothetical protein LOC680254 | 2.168 | 0.021 |
| Egfl6 | EGF-like-domain, multiple 6 | 2.168 | 0.029 |
| Rdh11 | retinol dehydrogenase 11 (all-trans/9-cis/11-cis) | 2.167 | 0.003 |
| Mki67 | marker of proliferation Ki-67 | 2.165 | 0.016 |
| Zfp566 | zinc finger protein 566 | 2.164 | 0.016 |
| Ebf1 | early B-cell factor 1 | 2.162 | 0.003 |
| Rbmx2 | RNA binding motif protein, X-linked 2 | 2.158 | 0.019 |
| Hsd17b4 | hydroxysteroid (17-beta) dehydrogenase 4 | 2.158 | 0.046 |
| Nln | neurolysin | 2.150 | 0.010 |
| Pik3c2g | phosphatidylinositol-4-phosphate 3-kinase, catalytic subunit type 2 gamma | 2.146 | 0.026 |
| Mapk6 | mitogen-activated protein kinase 6 | 2.142 | 0.039 |
| Prpf4b | pre-mRNA processing factor 4B | 2.139 | 0.047 |
| Zrsr1 | zinc finger (CCCH type), RNA binding motif and serine/arginine rich 1 | 2.137 | 0.003 |
| Upf3b | UPF3 regulator of nonsense transcripts homolog B (yeast) | 2.131 | 0.001 |
| Gnas | GNAS complex locus | 2.121 | 0.000 |
| Ifi47 | interferon gamma inducible protein 47 | 2.119 | 0.034 |
| Pf4 | platelet factor 4 | 2.116 | 0.026 |
| Rpl4 | ribosomal protein L4 | 2.114 | 0.009 |
| Vimp | VCP-interacting membrane selenoprotein | 2.110 | 0.024 |
| Clock | clock circadian regulator | 2.104 | 0.033 |
| Srek1ip1 | SREK1-interacting protein 1 | 2.101 | 0.012 |
| Alpl | alkaline phosphatase, liver/bone/kidney | 2.099 | 0.028 |
| Tpp2 | tripeptidyl peptidase 2 | 2.098 | 0.031 |
| Ankhd1 | ankyrin repeat and KH domain containing 1 | 2.097 | 0.016 |
| Cdk1 | cyclin-dependent kinase 1 | 2.094 | 0.006 |
| Tmem86a | transmembrane protein 86A | 2.092 | 0.043 |
| Hmgn5 | high mobility group nucleosome binding domain 5 | 2.086 | 0.032 |
| Pfn2 | profilin 2 | 2.082 | 0.025 |
| Ssbp3 | single stranded DNA binding protein 3 | 2.079 | 0.015 |
| Dcaf5 | DDB1 and CUL4 associated factor 5 | 2.079 | 0.032 |
| Cdh11 | cadherin 11 | 2.078 | 0.007 |
| Golph3l | golgi phosphoprotein 3-like | 2.078 | 0.011 |
| Nxpe3 | neurexophilin and PC-esterase domain family, member 3 | 2.075 | 0.021 |
| Slbp | stem-loop binding protein | 2.075 | 0.001 |
| Ltbp1 | latent transforming growth factor beta binding protein 1 | 2.070 | 0.021 |
| Brd4 | bromodomain containing 4 | 2.063 | 0.018 |
| Arcn1 | archain 1 | 2.060 | 0.010 |
| Akirin2 | akirin 2 | 2.056 | 0.008 |
| Mex3c | mex-3 RNA binding family member C | 2.056 | 0.045 |
| Cep83 | centrosomal protein 83 | 2.054 | 0.004 |
| Pdp2 | pyruvate dehyrogenase phosphatase catalytic subunit 2 | 2.050 | 0.042 |
| Armc8 | armadillo repeat containing 8 | 2.050 | 0.049 |
| Ccdc153 | coiled-coil domain containing 153 | 2.043 | 0.037 |
| Top1 | topoisomerase (DNA) I | 2.041 | 0.002 |
| Zfp384 | zinc finger protein 384 | 2.039 | 0.016 |
| Slc25a28 | solute carrier family 25 member 28 | 2.033 | 0.002 |
| Akr1cl | aldo-keto reductase family 1, member C-like | 2.030 | 0.016 |
| Slc39a6 | solute carrier family 39 member 6 | 2.026 | 0.032 |
| Smim14 | small integral membrane protein 14 | 2.026 | 0.024 |
| Herc4 | HECT and RLD domain containing E3 ubiquitin protein ligase 4 | 2.022 | 0.002 |
| Nudcd2 | NudC domain containing 2 | 2.013 | 0.038 |
| Cd44 | CD44 molecule (Indian blood group) | 2.007 | 0.017 |
| Birc3 | baculoviral IAP repeat-containing 3 | 2.007 | 0.011 |
| Ssfa2 | sperm specific antigen 2 | 2.001 | 0.012 |
| ***Downregulated genes*** | | | |
| Mthfd1l | methylenetetrahydrofolate dehydrogenase (NADP+ dependent) 1-like | 0.500 | 0.002 |
| Thsd1 | thrombospondin type 1 domain containing 1 | 0.500 | 0.005 |
| Snurf | SNRPN upstream reading frame | 0.500 | 0.014 |
| Sod3 | superoxide dismutase 3, extracellular | 0.499 | 0.038 |
| Idh2 | isocitrate dehydrogenase (NADP(+)) 2, mitochondrial | 0.499 | 0.008 |
| Gpsm1 | G-protein signaling modulator 1 | 0.498 | 0.031 |
| Josd2 | Josephin domain containing 2 | 0.498 | 0.028 |
| Bdh2 | 3-hydroxybutyrate dehydrogenase, type 2 | 0.497 | 0.003 |
| Cln8 | ceroid-lipofuscinosis, neuronal 8 | 0.497 | 0.027 |
| Glp1r | glucagon-like peptide 1 receptor | 0.496 | 0.007 |
| Spns2 | spinster homolog 2 | 0.495 | 0.003 |
| Crispld2 | cysteine-rich secretory protein LCCL domain containing 2 | 0.495 | 0.005 |
| Ttc19 | tetratricopeptide repeat domain 19 | 0.495 | 0.036 |
| Wfdc21 | WAP four-disulfide core domain 21 | 0.494 | 0.025 |
| Slc25a3 | solute carrier family 25 member 3 | 0.494 | 0.016 |
| Dusp14 | dual specificity phosphatase 14 | 0.493 | 0.004 |
| Lck | LCK proto-oncogene, Src family tyrosine kinase | 0.493 | 0.022 |
| Sepw1 | selenoprotein W, 1 | 0.493 | 0.016 |
| Nsmf | NMDA receptor synaptonuclear signaling and neuronal migration factor | 0.492 | 0.018 |
| Prelp | proline and arginine rich end leucine rich repeat protein | 0.492 | 0.016 |
| Slc25a25 | solute carrier family 25 member 25 | 0.491 | 0.010 |
| Elk1 | ELK1, ETS transcription factor | 0.490 | 0.027 |
| Sh2d3c | SH2 domain containing 3C | 0.489 | 0.005 |
| Alox5 | arachidonate 5-lipoxygenase | 0.489 | 0.006 |
| Mras | muscle RAS oncogene homolog | 0.488 | 0.000 |
| Lppr3 |  | 0.488 | 0.036 |
| Daxx | death-domain associated protein | 0.488 | 0.044 |
| Scarf2 | scavenger receptor class F, member 2 | 0.487 | 0.008 |
| Anks1a | ankyrin repeat and sterile alpha motif domain containing 1A | 0.487 | 0.022 |
| Rpusd1 | RNA pseudouridylate synthase domain containing 1 | 0.486 | 0.036 |
| Slc39a5 | solute carrier family 39 member 5 | 0.486 | 0.001 |
| Fbxl12 | F-box and leucine-rich repeat protein 12 | 0.485 | 0.017 |
| Fntb | farnesyltransferase, CAAX box, beta | 0.484 | 0.024 |
| Scamp4 | secretory carrier membrane protein 4 | 0.484 | 0.032 |
| Zfp467 | zinc finger protein 467 | 0.484 | 0.042 |
| Atp2a3 | ATPase sarcoplasmic/endoplasmic reticulum Ca2+ transporting 3 | 0.484 | 0.014 |
| Adh5 | alcohol dehydrogenase 5 (class III), chi polypeptide | 0.484 | 0.007 |
| Wif1 | Wnt inhibitory factor 1 | 0.483 | 0.000 |
| Mri1 | methylthioribose-1-phosphate isomerase 1 | 0.483 | 0.031 |
| Irf1 | interferon regulatory factor 1 | 0.482 | 0.035 |
| Marveld1 | MARVEL domain containing 1 | 0.482 | 0.005 |
| Hnrnpa1 | heterogeneous nuclear ribonucleoprotein A1 | 0.482 | 0.029 |
| Slc5a6 | solute carrier family 5 member 6 | 0.481 | 0.022 |
| Cabin1 | calcineurin binding protein 1 | 0.480 | 0.004 |
| Jund | JunD proto-oncogene, AP-1 transcription factor subunit | 0.480 | 0.003 |
| Ets2 | ETS proto-oncogene 2 | 0.479 | 0.000 |
| Srpx2 | sushi-repeat-containing protein, X-linked 2 | 0.478 | 0.027 |
| Ncdn | Neurochondrin | 0.478 | 0.026 |
| Col13a1 | collagen type XIII alpha 1 chain | 0.478 | 0.024 |
| Ddx56 | DEAD-box helicase 56 | 0.478 | 0.047 |
| Ccrl2 | C-C motif chemokine receptor like 2 | 0.477 | 0.018 |
| Rapgef3 | Rap guanine nucleotide exchange factor 3 | 0.477 | 0.020 |
| Pim3 | Pim-3 proto-oncogene, serine/threonine kinase | 0.476 | 0.041 |
| RGD1306151 |  | 0.476 | 0.026 |
| Cyp27a1 | cytochrome P450, family 27, subfamily a, polypeptide 1 | 0.476 | 0.029 |
| LOC361985 | similar to NICE-3 | 0.476 | 0.012 |
| Chst14 | carbohydrate sulfotransferase 14 | 0.475 | 0.002 |
| Tmem180 |  | 0.475 | 0.022 |
| Syne4 | spectrin repeat containing, nuclear envelope family member 4 | 0.475 | 0.033 |
| Trim47 | tripartite motif-containing 47 | 0.474 | 0.031 |
| Vhl | von Hippel-Lindau tumor suppressor | 0.474 | 0.007 |
| Tmem100 | transmembrane protein 100 | 0.472 | 0.009 |
| Ajuba | ajuba LIM protein | 0.470 | 0.005 |
| Mapk3 | mitogen activated protein kinase 3 | 0.467 | 0.001 |
| Rhoj | ras homolog family member J | 0.467 | 0.036 |
| Dok1 | docking protein 1 | 0.466 | 0.014 |
| Cactin | cactin, spliceosome C complex subunit | 0.465 | 0.001 |
| Tspo | translocator protein | 0.465 | 0.024 |
| Kif26b | kinesin family member 26B | 0.464 | 0.016 |
| Prmt2 | protein arginine methyltransferase 2 | 0.464 | 0.004 |
| Phf11 | PHD finger protein 11 | 0.463 | 0.042 |
| Nfe2 | nuclear factor, erythroid 2 | 0.462 | 0.022 |
| Apbb1 | amyloid beta precursor protein binding family B member 1 | 0.462 | 0.002 |
| Cd69 | Cd69 molecule | 0.461 | 0.024 |
| Tppp3 | tubulin polymerization-promoting protein family member 3 | 0.461 | 0.012 |
| Gadd45g | growth arrest and DNA-damage-inducible, gamma | 0.461 | 0.028 |
| Apol3 | apolipoprotein L, 3 | 0.459 | 0.018 |
| Gdf1 | growth differentiation factor 1 | 0.458 | 0.003 |
| Cers1 | ceramide synthase 1 | 0.458 | 0.003 |
| Enho | energy homeostasis associated | 0.458 | 0.017 |
| Bad | BCL2-associated agonist of cell death | 0.456 | 0.008 |
| Zfp219 | zinc finger protein 219 | 0.455 | 0.002 |
| Kctd10 | potassium channel tetramerization domain containing 10 | 0.455 | 0.012 |
| Sh2d2a | SH2 domain containing 2A | 0.455 | 0.019 |
| Fundc2 | FUN14 domain containing 2 | 0.454 | 0.004 |
| Mthfd1 | methylenetetrahydrofolate dehydrogenase, cyclohydrolase and formyltetrahydrofolate synthetase 1 | 0.453 | 0.018 |
| Dcakd | dephospho-CoA kinase domain containing | 0.452 | 0.048 |
| Ddx49 | DEAD-box helicase 49 | 0.452 | 0.018 |
| Zfp703 | zinc finger protein 703 | 0.451 | 0.007 |
| G4 | G4 protein | 0.449 | 0.029 |
| Phc2 | polyhomeotic homolog 2 | 0.449 | 0.005 |
| Mxra8 | matrix remodeling associated 8 | 0.448 | 0.003 |
| A3galt2 | alpha 1,3-galactosyltransferase 2 | 0.448 | 0.008 |
| Abcc10 | ATP binding cassette subfamily C member 10 | 0.447 | 0.032 |
| Fam127b | family with sequence similarity 127, member B | 0.447 | 0.007 |
| RGD1564482 | RGD1564482 | 0.447 | 0.002 |
| Sept1 | septin 1 | 0.446 | 0.016 |
| Raver2 | ribonucleoprotein, PTB-binding 2 | 0.446 | 0.042 |
| Itm2a | integral membrane protein 2A | 0.446 | 0.001 |
| Rras | related RAS viral (r-ras) oncogene homolog | 0.445 | 0.038 |
| Tpcn1 | two pore segment channel 1 | 0.445 | 0.004 |
| Thra | thyroid hormone receptor alpha, transcript variant TRalpha1 | 0.441 | 0.001 |
| Mgat4b | mannosyl (alpha-1,3-)-glycoprotein beta-1,4-N-acetylglucosaminyltransferase, isozyme B | 0.441 | 0.001 |
| Polg2 | polymerase (DNA) gamma 2, accessory subunit | 0.441 | 0.038 |
| Med22 | mediator complex subunit 22 | 0.440 | 0.023 |
| Cdc42ep4 | CDC42 effector protein 4 | 0.439 | 0.016 |
| Specc1l | sperm antigen with calponin homology and coiled-coil domains 1-like | 0.438 | 0.026 |
| Col6a2 | collagen, type VI, alpha 2 | 0.438 | 0.023 |
| Tgfb3 | transforming growth factor, beta 3 | 0.438 | 0.021 |
| Lyl1 | LYL1, basic helix-loop-helix family member | 0.438 | 0.018 |
| Syt5 | synaptotagmin 5 | 0.437 | 0.033 |
| Orai3 | ORAI calcium release-activated calcium modulator 3 | 0.437 | 0.006 |
| Slc9a1 | solute carrier family 9 member A1 | 0.436 | 0.035 |
| Alkbh6 | alkB homolog 6 | 0.435 | 0.015 |
| Camk1g | calcium/calmodulin-dependent protein kinase IG | 0.435 | 0.011 |
| Tbx1 | T-box 1 | 0.434 | 0.012 |
| Adgrl1 | adhesion G protein-coupled receptor L1 | 0.433 | 0.047 |
| Msra | methionine sulfoxide reductase A | 0.432 | 0.001 |
| Hlx | H2.0-like homeobox | 0.431 | 0.013 |
| Wfdc1 | WAP four-disulfide core domain 1 | 0.430 | 0.039 |
| C1qtnf5 | C1q and tumor necrosis factor related protein 5 | 0.430 | 0.038 |
| Nop16 | NOP16 nucleolar protein | 0.428 | 0.012 |
| Hps6 | Hermansky-Pudlak syndrome 6 | 0.427 | 0.010 |
| Fgfr4 | fibroblast growth factor receptor 4 | 0.427 | 0.023 |
| Fam65a | family with sequence similarity 65, member A | 0.427 | 0.015 |
| Ppdpf | pancreatic progenitor cell differentiation and proliferation factor | 0.426 | 0.014 |
| Abca7 | ATP binding cassette subfamily A member 7 | 0.426 | 0.001 |
| Plekhg5 | pleckstrin homology and RhoGEF domain containing G5 | 0.425 | 0.000 |
| Lama4 | laminin subunit alpha 4 | 0.425 | 0.007 |
| Ech1 | enoyl-CoA hydratase 1 | 0.425 | 0.024 |
| Ccdc97 | coiled-coil domain containing 97 | 0.425 | 0.003 |
| Rps6ka2 | ribosomal protein S6 kinase polypeptide 2 | 0.424 | 0.015 |
| Il17re | interleukin 17 receptor E | 0.423 | 0.002 |
| Arsb | arylsulfatase B | 0.423 | 0.035 |
| Exoc3l1 | exocyst complex component 3-like 1 | 0.423 | 0.037 |
| Arhgef18 | Rho/Rac guanine nucleotide exchange factor 18 | 0.423 | 0.006 |
| Sec22a | SEC22 homolog A, vesicle trafficking protein | 0.422 | 0.011 |
| Stmn4 | stathmin 4 | 0.422 | 0.012 |
| Abo | ABO blood group (transferase A, alpha 1-3-N-acetylgalactosaminyltransferase; transferase B, alpha 1-3-galactosyltransferase) | 0.421 | 0.010 |
| Cish | cytokine inducible SH2-containing protein | 0.420 | 0.006 |
| Csrnp1 | cysteine and serine rich nuclear protein 1 | 0.420 | 0.048 |
| Fanca | Fanconi anemia, complementation group A | 0.419 | 0.003 |
| Pigq | phosphatidylinositol glycan anchor biosynthesis, class Q | 0.419 | 0.003 |
| Pdk2 | pyruvate dehydrogenase kinase 2 | 0.419 | 0.015 |
| Fggy | FGGY carbohydrate kinase domain containing | 0.418 | 0.003 |
| Il4r | interleukin 4 receptor | 0.417 | 0.014 |
| Rbfa | ribosome binding factor A | 0.416 | 0.023 |
| Mdp1 | magnesium-dependent phosphatase 1 | 0.415 | 0.040 |
| Phf19 | PHD finger protein 19 | 0.413 | 0.035 |
| Crocc | ciliary rootlet coiled-coil, rootletin | 0.412 | 0.023 |
| Nap1l4 | nucleosome assembly protein 1-like 4 | 0.412 | 0.007 |
| Inpp5a | inositol polyphosphate-5-phosphatase A | 0.411 | 0.004 |
| Phf11b |  | 0.411 | 0.018 |
| Ptpn23 | protein tyrosine phosphatase, non-receptor type 23 | 0.411 | 0.013 |
| Sept4 | septin 4 | 0.410 | 0.042 |
| Armcx2 | armadillo repeat containing, X-linked 2 | 0.410 | 0.023 |
| Uaca | uveal autoantigen with coiled-coil domains and ankyrin repeats | 0.409 | 0.025 |
| Bcl6b | B-cell CLL/lymphoma 6B | 0.408 | 0.005 |
| Slc38a5 | solute carrier family 38, member 5 | 0.408 | 0.003 |
| Anxa2 | annexin A2 | 0.407 | 0.004 |
| Nacc2 | NACC family member 2 | 0.407 | 0.009 |
| Cldn3 | claudin 3 | 0.407 | 0.008 |
| Serpinb6 | serpin family B member 6 | 0.405 | 0.010 |
| Tbc1d25 | TBC1 domain family, member 25 | 0.403 | 0.015 |
| Hdac8 | histone deacetylase 8 | 0.401 | 0.032 |
| Bbc3 | Bcl-2 binding component 3 | 0.399 | 0.006 |
| Vtn | Vitronectin | 0.399 | 0.003 |
| Pskh1 | protein serine kinase H1 | 0.398 | 0.002 |
| Plekhg2 | pleckstrin homology and RhoGEF domain containing G2 | 0.398 | 0.013 |
| Plvap | plasmalemma vesicle associated protein | 0.396 | 0.029 |
| Rbp1 | retinol binding protein 1 | 0.395 | 0.028 |
| Wdr25l |  | 0.392 | 0.027 |
| Scn3b | sodium voltage-gated channel beta subunit 3 | 0.392 | 0.007 |
| Mfap5 | microfibrillar associated protein 5 | 0.391 | 0.047 |
| LOC103692984 | uncharacterized LOC103692984 | 0.391 | 0.040 |
| Rps6ka4 | ribosomal protein S6 kinase A4 | 0.391 | 0.017 |
| Krt19 | keratin 19 | 0.391 | 0.006 |
| Dok3 | docking protein 3 | 0.391 | 0.038 |
| Tjp3 | tight junction protein 3 | 0.390 | 0.011 |
| Sh3bgrl3 | SH3 domain binding glutamate-rich protein like 3 | 0.390 | 0.005 |
| Mfng | MFNG O-fucosylpeptide 3-beta-N-acetylglucosaminyltransferase | 0.389 | 0.024 |
| Mib2 | mindbomb E3 ubiquitin protein ligase 2 | 0.387 | 0.047 |
| Hspb1 | heat shock protein family B (small) member 1 | 0.386 | 0.003 |
| Rgs12 | regulator of G-protein signaling 12 | 0.382 | 0.023 |
| Ptp4a3 | protein tyrosine phosphatase type IVA, member 3 | 0.381 | 0.002 |
| Epb41l4a | erythrocyte membrane protein band 4.1 like 4A | 0.381 | 0.022 |
| Nabp2 | nucleic acid binding protein 2 | 0.380 | 0.016 |
| Cracr2b | calcium release activated channel regulator 2B | 0.379 | 0.003 |
| Ninj2 | ninjurin 2 | 0.378 | 0.044 |
| Fam26f | family with sequence similarity 26, member F | 0.378 | 0.030 |
| Serhl2 | serine hydrolase-like 2 | 0.377 | 0.019 |
| Ccdc126 | coiled-coil domain containing 126 | 0.377 | 0.031 |
| Tcn2 | transcobalamin 2 | 0.376 | 0.002 |
| Gsk3a | glycogen synthase kinase 3 alpha | 0.376 | 0.018 |
| Ahsp | alpha hemoglobin stabilizing protein | 0.376 | 0.000 |
| Bre | brain and reproductive organ-expressed (TNFRSF1A modulator) | 0.375 | 0.015 |
| Tpra1 | transmembrane protein adipocyte associated 1 | 0.374 | 0.006 |
| Kifc3 | kinesin family member C3 | 0.373 | 0.021 |
| Acta2 | actin, alpha 2, smooth muscle, aorta | 0.373 | 0.027 |
| Copz2 | coatomer protein complex, subunit zeta 2 | 0.371 | 0.015 |
| Tmem119 | transmembrane protein 119 | 0.370 | 0.006 |
| B3galt5 | Beta-1,3-galactosyltransferase 5 | 0.370 | 0.037 |
| Wnt2b | wingless-type MMTV integration site family, member 2B | 0.370 | 0.014 |
| Slc35c2 | solute carrier family 35 member C2 | 0.369 | 0.007 |
| Pin1 | peptidylprolyl cis/trans isomerase, NIMA-interacting 1 | 0.368 | 0.046 |
| Fmod | Fibromodulin | 0.368 | 0.014 |
| Ifrd2 | interferon-related developmental regulator 2 | 0.367 | 0.036 |
| Ccnjl | cyclin J-like | 0.367 | 0.013 |
| Npr1 | natriuretic peptide receptor 1 | 0.365 | 0.002 |
| Actc1 | actin, alpha, cardiac muscle 1 | 0.364 | 0.017 |
| Fam101a | family with sequence similarity 101, member A | 0.363 | 0.018 |
| Cldn12 | claudin 12 | 0.363 | 0.008 |
| Tmem59l | transmembrane protein 59-like | 0.362 | 0.039 |
| Slc4a3 | solute carrier family 4 member 3 | 0.360 | 0.001 |
| Itga7 | integrin subunit alpha 7 | 0.359 | 0.017 |
| Tmem259 | transmembrane protein 259 | 0.356 | 0.012 |
| Cdc42ep2 | CDC42 effector protein 2 | 0.356 | 0.021 |
| Zfp282 | zinc finger protein 282 | 0.353 | 0.036 |
| Alkbh4 | alkB homolog 4, lysine demethylase | 0.352 | 0.040 |
| Apba3 | amyloid beta precursor protein binding family A member 3 | 0.351 | 0.006 |
| Aplnr | apelin receptor | 0.351 | 0.017 |
| Acot1 | acyl-CoA thioesterase 1 | 0.351 | 0.044 |
| Fendrr | FOXF1 adjacent non-coding developmental regulatory RNA | 0.351 | 0.029 |
| Tpst2 | tyrosylprotein sulfotransferase 2 | 0.351 | 0.004 |
| Rbp4 | retinol binding protein 4 | 0.351 | 0.003 |
| Plcg2 | phospholipase C, gamma 2 | 0.350 | 0.037 |
| Ccl5 | C-C motif chemokine ligand 5 | 0.350 | 0.015 |
| Fcnb | ficolin B | 0.349 | 0.016 |
| Fam89a | family with sequence similarity 89, member A | 0.349 | 0.019 |
| RGD1564379 | RGD1564379 | 0.345 | 0.014 |
| Txk | TXK tyrosine kinase | 0.343 | 0.018 |
| Sh3bgr | SH3 domain binding glutamate-rich protein | 0.342 | 0.014 |
| Ddit4 | DNA-damage-inducible transcript 4 | 0.342 | 0.005 |
| Actr5 | ARP5 actin-related protein 5 homolog | 0.342 | 0.012 |
| Dhx35 | DEAH-box helicase 35 | 0.341 | 0.019 |
| Cyb561a3 | cytochrome b561 family, member A3 | 0.341 | 0.037 |
| Tmcc2 | transmembrane and coiled-coil domain family 2 | 0.338 | 0.027 |
| S100a9 | S100 calcium binding protein A9 | 0.338 | 0.012 |
| LOC100912041 | uncharacterized LOC100912041 | 0.338 | 0.031 |
| Echdc3 | enoyl CoA hydratase domain containing 3 | 0.336 | 0.046 |
| Mdfi | MyoD family inhibitor | 0.335 | 0.026 |
| LOC24906 | RoBo-1 | 0.334 | 0.009 |
| Tbx2 | T-box 2 | 0.332 | 0.002 |
| Amigo2 | adhesion molecule with Ig like domain 2 | 0.331 | 0.012 |
| Gemin7 | gem (nuclear organelle) associated protein 7 | 0.324 | 0.032 |
| Pcolce | procollagen C-endopeptidase enhancer | 0.320 | 0.003 |
| Pex11g | peroxisomal biogenesis factor 11 gamma | 0.316 | 0.030 |
| Eln | Elastin | 0.312 | 0.031 |
| Tmem116 | transmembrane protein 116 | 0.312 | 0.010 |
| Cxcl14 | C-X-C motif chemokine ligand 14 | 0.310 | 0.021 |
| Nckap5l | NCK-associated protein 5-like | 0.308 | 0.006 |
| Grin3b | glutamate ionotropic receptor NMDA type subunit 3B | 0.308 | 0.013 |
| Rab15 | RAB15, member RAS oncogene family | 0.303 | 0.011 |
| Ptgis | prostaglandin I2 (prostacyclin) synthase | 0.303 | 0.004 |
| Fam83f | family with sequence similarity 83, member F | 0.302 | 0.049 |
| Gckr | glucokinase (hexokinase 4) regulator | 0.301 | 0.008 |
| Rnase2 | ribonuclease A family member 2 | 0.300 | 0.013 |
| Peli3 | pellino E3 ubiquitin protein ligase family member 3 | 0.296 | 0.043 |
| Ngef | neuronal guanine nucleotide exchange factor | 0.293 | 0.041 |
| Tmem246 | transmembrane protein 246 | 0.290 | 0.046 |
| Gzmm | granzyme M | 0.289 | 0.005 |
| Art2b | ADP-ribosyltransferase 2b | 0.287 | 0.011 |
| Gpr132 | G protein-coupled receptor 132 | 0.280 | 0.013 |
| Lipe | lipase E, hormone sensitive type | 0.278 | 0.013 |
| Pgf | placental growth factor | 0.274 | 0.009 |
| Mrpl4 | mitochondrial ribosomal protein L4 | 0.271 | 0.002 |
| Nov | nephroblastoma overexpressed | 0.271 | 0.019 |
| Tp53i13 | tumor protein p53 inducible protein 13 | 0.266 | 0.032 |
| Dlg4 | discs large MAGUK scaffold protein 4 | 0.265 | 0.039 |
| Ikbip | IKBKB interacting protein | 0.264 | 0.023 |
| Adamts9 | ADAM metallopeptidase with thrombospondin type 1 motif, 9 | 0.264 | 0.011 |
| Tll1 | tolloid-like 1 | 0.262 | 0.008 |
| Fos | FBJ osteosarcoma oncogene | 0.254 | 0.024 |
| Stac2 | SH3 and cysteine rich domain 2 | 0.254 | 0.035 |
| Ier2 | immediate early response 2 | 0.253 | 0.024 |
| Snph | syntaphilin | 0.251 | 0.024 |
| Rprml | reprimo-like | 0.248 | 0.015 |
| Sit1 | signaling threshold regulating transmembrane adaptor 1 | 0.248 | 0.011 |
| Ear11 | eosinophil-associated, ribonuclease A family, member 11 | 0.239 | 0.007 |
| Kif5c | kinesin family member 5C | 0.239 | 0.014 |
| Twist2 | twist family bHLH transcription factor 2 | 0.232 | 0.016 |
| Emid1 | EMI domain containing 1 | 0.229 | 0.022 |
| Igfbpl1 | insulin-like growth factor binding protein-like 1 | 0.228 | 0.015 |
| Rrad | RRAD, Ras related glycolysis inhibitor and calcium channel regulator | 0.226 | 0.018 |
| Slc6a4 | solute carrier family 6 member 4 | 0.226 | 0.000 |
| Cpa2 | carboxypeptidase A2 | 0.224 | 0.039 |
| Foxe1 | forkhead box E1 | 0.222 | 0.039 |
| Mpp3 | membrane palmitoylated protein 3 | 0.216 | 0.012 |
| Abcg4 | ATP binding cassette subfamily G member 4 | 0.207 | 0.005 |
| Ltc4s | leukotriene C4 synthase | 0.206 | 0.025 |
| Mmp28 | matrix metallopeptidase 28 | 0.205 | 0.005 |
| S100a3 | S100 calcium binding protein A3 | 0.204 | 0.010 |
| Ngp | neutrophilic granule protein | 0.196 | 0.010 |
| Heyl | hes-related family bHLH transcription factor with YRPW motif-like | 0.196 | 0.012 |
| Myo1a | myosin IA | 0.196 | 0.017 |
| Deaf1 | DEAF1 transcription factor | 0.187 | 0.001 |
| Fcho1 | FCH domain only 1 | 0.187 | 0.033 |
| Lynx1 | Ly6/neurotoxin 1 | 0.185 | 0.035 |
| Clec2l | C-type lectin domain family 2, member L | 0.171 | 0.001 |
| S100a8 | S100 calcium binding protein A8 | 0.170 | 0.022 |
| Slc1a7 | solute carrier family 1 member 7 | 0.169 | 0.029 |
| Dlk2 | delta like non-canonical Notch ligand 2 | 0.152 | 0.007 |
| Dusp9 | dual specificity phosphatase 9 | 0.150 | 0.018 |
| Pdia5 | protein disulfide isomerase family A, member 5 | 0.140 | 0.015 |
| Bcl9l | B-cell CLL/lymphoma 9-like | 0.125 | 0.020 |
| Fam25a | family with sequence similarity 25, member A | 0.119 | 0.000 |
| Slc26a3 | solute carrier family 26 member 3 | 0.114 | 0.036 |
| Siglec5 | sialic acid binding Ig-like lectin 5 | 0.068 | 0.003 |
